# An Investigation of the Constructional Design Components Affecting the Mechanical Response and Cellular Activity of Electrospun Vascular Grafts

**DOI:** 10.3390/membranes12100929

**Published:** 2022-09-25

**Authors:** Suzan Ozdemir, Ipek Yalcin-Enis, Baturalp Yalcinkaya, Fatma Yalcinkaya

**Affiliations:** 1 Textile Engineering Department, Textile Technologies and Design Faculty, Istanbul Technical University, Beyoglu, 34467 Istanbul, Turkey; 2 Department of Material Science, Faculty of Mechanical Engineering, Technical University of Liberec, 461 17 Liberec, Czech Republic; 3 Department of Environmental Technology, Institute for Nanomaterials, Advanced Technologies and Innovations, Technical University of Liberec, 461 17 Liberec, Czech Republic

**Keywords:** vascular grafts, biopolymers, physiological forces, compliance, burst pressure, cellular activity, permeability, porosity, fiber orientation, wall thickness

## Abstract

Cardiovascular disease is anticipated to remain the leading cause of death globally. Due to the current problems connected with using autologous arteries for bypass surgery, researchers are developing tissue-engineered vascular grafts (TEVGs). The major goal of vascular tissue engineering is to construct prostheses that closely resemble native blood vessels in terms of morphological, mechanical, and biological features so that these scaffolds can satisfy the functional requirements of the native tissue. In this setting, morphology and cellular investigation are usually prioritized, while mechanical qualities are generally addressed superficially. However, producing grafts with good mechanical properties similar to native vessels is crucial for enhancing the clinical performance of vascular grafts, exposing physiological forces, and preventing graft failure caused by intimal hyperplasia, thrombosis, aneurysm, blood leakage, and occlusion. The scaffold’s design and composition play a significant role in determining its mechanical characteristics, including suturability, compliance, tensile strength, burst pressure, and blood permeability. Electrospun prostheses offer various models that can be customized to resemble the extracellular matrix. This review aims to provide a comprehensive and comparative review of recent studies on the mechanical properties of fibrous vascular grafts, emphasizing the influence of structural parameters on mechanical behavior. Additionally, this review provides an overview of permeability and cell growth in electrospun membranes for vascular grafts. This work intends to shed light on the design parameters required to maintain the mechanical stability of vascular grafts placed in the body to produce a temporary backbone and to be biodegraded when necessary, allowing an autologous vessel to take its place.

## 1. Introduction

Cardiovascular diseases (CVDs) remain the major cause of death worldwide, and an estimated 17.9 million individuals died in 2019. Additionally, prior CVDs are a significant risk factor for coronavirus disease of 2019 (COVID-19)-related complications and fatalities [1,2]. The main risk factors for CVD include smoking, being overweight, having diabetes, high blood pressure or hypertension, dyslipidemia, not exercising enough, eating poorly, and experiencing a lot of stress, all of which are extremely prevalent problems in today’s society [3,4]. The number of CVD cases increased from 271 million in 1990 to 523 million in 2019, while CVD fatalities increased from 12.1 million in 1990 to 18.6 million in 2019 [5]. The World Health Organization estimates that by 2030, there will be a 24.5% increase in the number of fatalities [6]. Coronary artery disease, the most prevalent form of CVD, necessitates surgery based on arterial replacement, known as bypass grafting [7]. The blood vessel that is injured or obstructed is replaced during bypass surgeries with an autologous vein or synthetic graft. Autologous grafts have significant disadvantages because of their scarcity and difficulties with graft harvesting [8]. Despite being the most common autograft, the saphenous vein has low patency and a failure rate of about 50% after ten years of implantation [9]. Vascular grafts made of synthetic materials can be used in place of autologous vessels. Expanded polytetrafluoroethylene (ePTFE, Gore-Tex, California, USA) and polyethylene terephthalate (PET, Dacron, Invista, Kansas, USA) are the most widely used commercial synthetic materials because they are effective at replacing large-diameter arteries and have shown successful long-term results. However, they are ineffective when used as smaller diameter vascular grafts (<6 mm), such as coronary arteries, because of low patency rates, thrombogenicity, and compliance mismatch [10,11]. The compliance mismatch between the native artery and the inelastic synthetic graft at the anastomosis sites results in low blood flow rates and turbulent blood flow in small-diameter grafts.

Due to these mechanical issues, the thrombogenic nature of the scaffold material, poor endothelialization, luminal narrowing, and thrombosis are brought on by intimal hyperplasia, causing low patency rates [12]. Enhancing the mechanical performance and biocompatibility of small-caliber vascular prostheses is necessary to satisfy a clinical requirement and offer patients alternative scaffolds due to the current limitations of clinically approved grafts [13]. However, the clinical applicability of vascular grafts is still constrained by problems with the intrinsic thrombogenic character of synthetic polymers, inability to sustain somatic growth and repair, inappropriate mechanical qualities, and severe intimal hyperplasia [14]. Thus, novel approaches for fabricating TEVGs, including electrospinning, decellularization, lyophilization, and 3D printing by utilizing biopolymers, have been explored to eliminate these issues and provide the ideal small-caliber graft that may be used in the clinic and can imitate the native artery in all aspects [15]. The selection of the material and the production technique is based on the determination and optimization of the design parameters, which require a better understanding of the vascular environment, the properties and needs of native vessels, and the correlation between the constructional criteria and graft properties. In this regard, this review covers vascular grafts, scaffold fabrication methods, biopolymers utilized in these prostheses, and mechanical forces acting on vascular grafts in detail. In addition, the impact of constructional design parameters on mechanical as well as permeability properties is discussed, and recent studies have been reviewed in the literature to give a broad perspective for the researchers to discover the necessities and limitations in this field and help to find alternative ways to meet the requirements of vascular grafts improved in the future.

## 2. Anatomy of Blood Vessels

**Figure 1:**
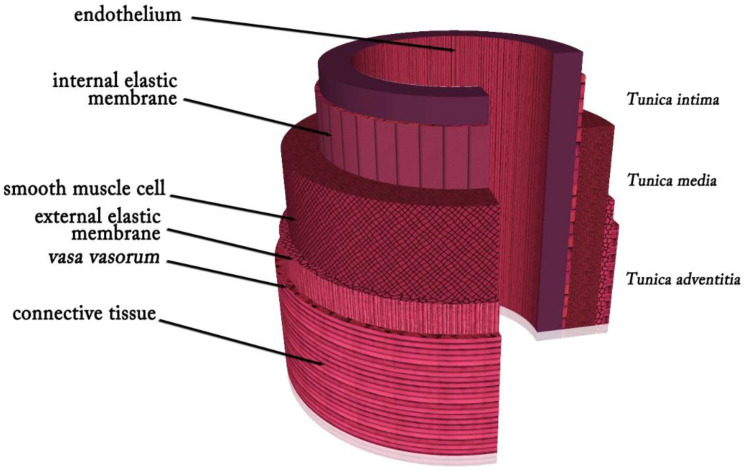
Layers of an artery.

Arteries, capillaries, and veins are all linked in series to make up the pulmonary vasculature [16]. Arteries and veins accomplish effective blood circulation across the lumen to distant locations. Arteries transport oxygenated blood from the heart to the tissues, whereas veins transport waste, nutrients, and oxygen from the capillaries back to the heart while returning deoxygenated blood to it [17,18]. The extracellular matrix (ECM) comprises 70% water, and the remaining 30% comprises a vascular wall consisting of collagen, elastin, proteoglycans, and vascular cells [19]. The three layers that form the typical arterial wall are the *tunica intima*, which consists of a single layer of endothelial cells (ECs) that exists in the internal elastic *lamina*, which is a dense elastic membrane that divides the *intima* from the *media* and is oriented parallel to the blood flow; the *tunica media*, which is composed of concentric layers of smooth muscle cells (SMCs) between the elastic *lamina* layers; and the *tunica adventitia*, which is formed of myofibroblasts involving connective tissue with nerve fibers and the *vasa vasorum* that nurtures the blood vessel wall and is divided from the *media* by an external elastic *lamina* [20,21] (Figure 1). The *tunica intima*, also known as the endothelium layer, controls the tone of blood vessels, platelet activation, adhesion and aggregation, leukocyte adherence, SMC migration, and proliferation and serves as a thrombo-resistant, continuous selective permeable wall that permits laminar blood flow throughout the blood vessel [22]. ECs and SMCs play crucial roles in preserving the vessel’s mechanical efficiency and structural integrity. The *tunica media*, in which collagen, elastin fibers, and SMCs are radially aligned, offers the vessel mechanical strength and regulates vessel diameter by contracting or relaxing [23]. High blood pressure causes the arteries to experience significant mechanical stress. In the physiological pressure range, the load on the vessel is distributed between the collagen and elastin fibers. At higher blood pressures, where a greater amount of force is needed for a change in diameter, the stiffer collagen fibers dominate the mechanical behavior and protect the blood vessel from failure. In contrast, the elastic components, which are less stiff and more elastic chains, dominate mechanical behavior at lower pressures [19]. Collagen fibers, elastic fibers, elastic lamellae, and proteoglycans, which provide vessel elasticity and radial compliance, are secreted by SMCs [22]. The flexibility and structural stability of the artery are supported by elastic *laminae*. Intimal hyperplasia is avoided thanks to elastic fibers and *lamina* that slow down SMC development [24]. Elastin, which relieves stress on the heart and permits vasodilation and vasoconstriction in arteries with pulsatile flow, is responsible for reversible elasticity [25]. The *vasa vasorum* and vascular innervation are supported by the *tunica adventitia*. This outermost layer comprises fibroblasts, extracellular matrix, and fibrillar types I and III collagen and is placed between the exterior elastic *lamina* on the *media* layer and interstitial matrix [23,24].

## 3. Requirements for Vascular Grafts

The fundamental concern with vascular tissue engineering is still creating an ideal vascular graft that can replicate the structural, biological, and mechanical characteristics of the native blood vessels and be used as a replacement for the damaged blood vessel. When selecting a polymer and a method of fabrication for the construction of synthetic blood vessels, some fundamental properties to consider are processability, mechanical behavior, morphology and porosity, hydrophilicity, biodegradability, and biocompatibility [26]. The vascular scaffolds should offer an ideal topographic and structural framework for cell adhesion, proliferation, and diffusion [27]. Developing a suitable microstructure and functional material that encourages endothelialization requires proper processing and surface modification techniques. Additionally, the native tissues must be compatible with the mechanical characteristics of small-diameter vascular grafts, including modulus, nonlinear elasticity, compliance, burst pressure, and suture retention strength, as even a slight mechanical discrepancy between the prostheses and the native vessel can lead to graft failure [28]. A combination of dynamic mechanical forces, including hemodynamic forces originating from fluid flow, cyclic stretch, lateral pressure, and vessel wall forces created by the vasculature, creates the complex mechanical microenvironment [29]. The compliance mismatch, which is the incompatible dimensional change of the vascular graft and the native blood vessel in response to pressure variations inside the lumen, causes the hemodynamic flow imbalance in the vascular graft and stress concentration at the anastomosis. The pressure difference at the anastomosis can result in intimal hyperplasia and thrombosis [30]. Additionally, just like the native artery, the designed blood vessel’s burst pressure must be higher than 1000 mmHg in order to resist blood pressure (200 mmHg) [31].

The pore size is a crucial structural element since it directly impacts cell migration, leakproofness, and mechanical qualities. Large pores obviously cause blood leakage, but small pores prevent cells from penetrating. SMCs must enter TEVGs through larger pores since they are bigger than ECs [32]. Additionally, in vitro research on the development of the macrovascular endothelium has shown that materials with smaller pore diameters and lower porosity promote better EC adherence. ECs range from 10 to 40 μm, and adhesion is necessary for their proliferation. As a result, the proliferation is limited to materials with pores larger than a cell, particularly those with diameters of more than 30 μm [33]. Moreover, a reduction in mechanical characteristics is observed with an increase in pore size and porosity [34]. In addition, vascular scaffolds should be suturable for convenience of use, and for usage in ophthalmic and microvascular procedures, a 0.6 N suture retention is adequate [35,36]. The choice of materials for vascular grafts is also heavily influenced by biodegradation. A fast degradation rate can limit neointimal hyperplasia by limiting the activation of inflammatory cells and reducing the probability and intensity of a foreign body response. However, a high rate of tissue degradation can compromise its performance. Thus, the vascular tissue regeneration rate should be harmonious with the biodegradation rate [37].

The topography and morphology of the lumen surface are other essential aspects affecting thrombosis and material selection. In addition, gradients strongly influence platelet adhesion and activation in the nanotopography of the material surface. Thus, a TEVG surface designed with the proper topography and roughness could significantly increase the hemocompatibility of the lumen surface of the scaffold [38].

Thus, for obtaining an applicable small-diameter vascular graft that satisfies all of the necessary features, this scaffold needs to be mechanically strong and compliant to withstand hemodynamic stress; suturable; available in various sizes in case of emergency; easy to use to reduce the time, cost, and risk; resistant to thrombus and infection; biocompatible to integrate with the body and allow the formation of neo-vessels similar to native arteries in characteristics and performance; low-cost; patent for long-term; able to show rapid endothelialization; and porous enough for easy cell diffusion; it is necessary to combine biomimetic design with improved cellular and molecular knowledge of the biology of the vessel wall [39].

## 4. Electrospinning Technique

The ability to have sufficient multicellular activities, nutrition delivery, and mechanical qualities is essential to successfully produce scaffolds at the macro- and microscales [40]. Solvent casting with particle leaching, thermally induced phase separation, freeze drying, electrospinning, 3D printing, and combination molding techniques are some methods for constructing tissue-engineered scaffolds that imitate the ECM [41]. Among these methods, electrospinning has emerged as a leading technology for creating synthetic polymer grafts because it is configurable, and the electrospun fibrous structure closely resembles the fibrous structure of native ECM in the vessel wall, enabling cell infiltration and cellularization of the grafts and having a high surface-to-volume ratio thanks to 3D fibrous matrices with varying fiber sizes [42].

**Figure 2:**
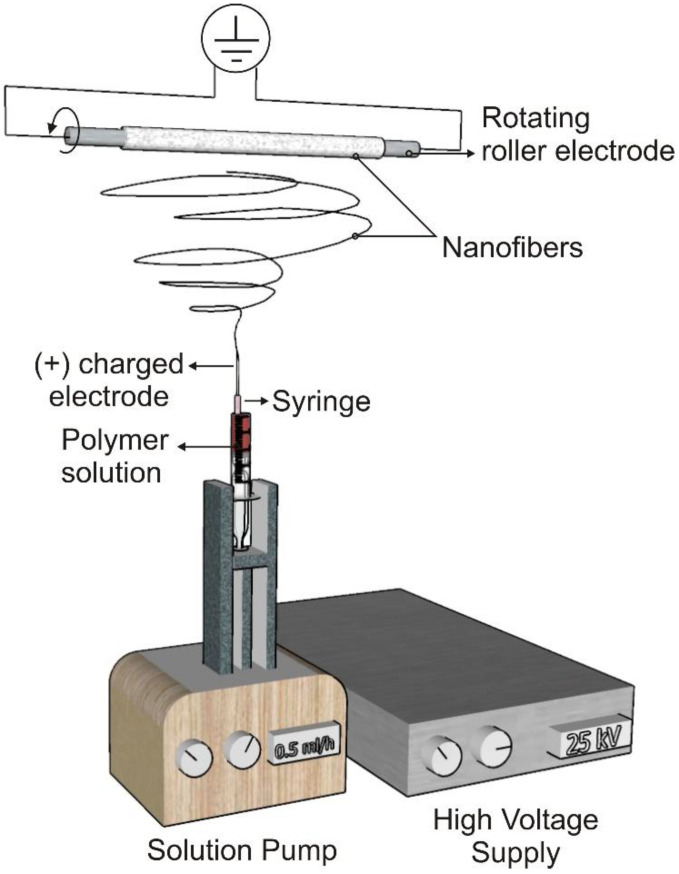
Electrospinning set-up.

Nanoscale (<1000 nm) and microscale (>1 μm) polymer fibers can be created using the electrospinning technique [43]. The electrospinning setup consists of three main parts: a high voltage supplier, a capillary tube with a tip, and a collector [27] (Figure 2). When a voltage is applied during electrospinning, the electrostatic force affecting the droplet overcomes the surface tension and forces a liquid jet to move out from the tip. This whipping action results in the drawing and thinning of the polymer and causes fiber production as the solvent evaporates simultaneously. The geometry of the resultant scaffold is influenced by the collector type and electrospinning arrangement [44]. Electrospinning can be used to create biomimetic degradable scaffolds for essential cellular and molecular activities using both natural and synthetic biopolymers [45].

To encourage capillary ingrowth and graft regeneration, vascular grafts must have an acceptable level of porosity, with an average pore diameter of 10 μm and a minimum pore area of 20–80 μm^2^. Macrophages, cells, fibroblasts, and capillaries deposit on the wall with pore sizes of 25–40 μm and higher in the first few weeks, allowing cellular infiltration. Electrospinning may be a useful technology among other manufacturing processes for creating an appropriate microstructure for full neovessel production because of its ability to adjust fiber and pore diameters [20]. As a result, electrospun fibrous scaffolds have become much more promising candidates for vascular tissue engineering due to their highly porous structure and high surface-to-volume ratios, which encourage cell interactions, including EC adhesion and SMC diffusion into the porous outer layer [46,47].

The electrospinning parameters can be divided into three groups, which are solution parameters (viscosity, conductivity, molecular weight, and surface tension), process parameters (voltage, tip to collector distance, and flow rate), and ambient parameters (humidity and temperature) [48]. In order to produce scaffolds satisfying the requirements structurally, mechanically, and biologically, the optimum parameters must be chosen in accordance with the needs of the electrospinning method and the final prosthesis.

## 5. Design Components for Electrospun Vascular Prosthesis

The design parameters for electrospun vascular grafts can be divided into two categories: the constructional parameters, which involve fiber diameter, pore size, porosity, fiber orientation, wall thickness, the number of layers, and material selection. The scaffold’s configuration and material choice are both essential because they have a significant impact on mechanical and biological characteristics, including compliance, tensile strength, burst pressure, blood permeability, and suturability, as well as biological processes such as cell phenotype, ECM formation, and cell diffusion.

### 5.1. Constructional Parameters

It is challenging to design ideal 3D scaffolds that replicate the properties of ECM; thus, electrospinning is becoming more popular for making vascular grafts due to its potential to create scaffolds with micro/nano-scale topography, high surface area-to-volume proportions, and highly interconnected pores. Researchers can optimize the properties of prostheses and produce scaffolds with higher cell infiltration and proliferation and adequate mechanical properties by modifying the construction parameters of fibrous scaffolds by altering the electrospinning parameters [49].

#### 5.1.1. Fiber Diameter, Pore Size, Porosity, and Permeability

Ideal scaffolds are frequently fabricated to be very porous for cell diffusion, nutrient and oxygen delivery, and metabolic disposal of wastes to promote the development of targeted neotissues [50]. Small pore sizes are the major issue concerning electrospinning because they result in inadequate cell penetration and compliance mismatch [31,51]. This issue can be resolved by regulating porosity using various techniques, including salt/polymer leaching, collector modification, post-treatment with laser radiation, and adjusting the electrospinning conditions. It has been demonstrated that the pore size of electrospun webs is directly associated with the fiber diameter, suggesting that the pore size increases with an increase in fiber diameter. Thus, the diameter of the fiber can be easily modified by changing electrospinning variables such as the polymer concentration, voltage, and solvent type [51]. Even though electrospun prostheses made of nanofibers have a greater capacity for cell adhesion and proliferation than scaffolds made of microfibers, they frequently have lower cell infiltration levels. This is typically due to the small pore sizes, complex distribution, and lack of pore connectivity of scaffolds made of nanofibers, which have an impact on long-term matrix regeneration. Thus, using microfibers and nanofibers together encourages cell adhesion and proliferation with the help of nanofibers and gives more void areas for cell penetration through less dense microfibers [52].

The pore size of vascular grafts is recognized as an essential design parameter in the production of TEVGs because the vascular cells must be effectively settled with ECs on the lumen surface and SMCs in the outer layers. While ECs on the luminal surface prevent thrombosis, SMCs on the outer wall of vascular scaffolds support the scaffolds’ activities such as vasoconstriction and vasodilatation [53]. It is claimed that electrospun scaffolds with fiber diameters of more than 1 μm allow larger pore diameters and encourage cell penetration, whereas smaller fiber diameters of less than 1 μm dramatically restrict diffusion for the majority of cell types, and so the ideal pore diameter necessary for sufficient cellular penetration is greater than 10 μm [53,54,55]. Small pore diameters are acceptable for the ECs to accumulate, proliferate, and infiltrate on the graft surface, which encourages ECM regeneration; however, they hinder SMCs’ infiltration and colonization around the neo-vessel [53]. It has also been stated that the optimum scaffold pore diameter ranges from 5 to 500 μm since distinct cell types have unique dimensions and morphologies [56,57]. Large pores are ideal for better cell diffusion but can also promote blood leakage through the graft wall. With a homogeneous design, it is challenging to achieve a balance among enhanced tissue regeneration, decreased blood leakage, and sufficient mechanical characteristics; for this reason, multilayered vascular prostheses with different pore diameters have been thought to be useful [56]. Additionally, it is claimed that grafts with a porosity of 90% and pore sizes between 100 and 300 μm can effectively support cell adhesion and matrix development. When SMCs are cultured on these scaffolds, the mechanical behavior can be changed from elastic to viscoelastic, more closely approximating the mechanical characteristics of the native vessels [58].

The mechanical characteristics of the vascular scaffolds are also greatly influenced by the fiber diameter, pore size, and porosity, in addition to their biological impacts. Fluid permeability, thermal conductivity, diffusion coefficient, elastic modulus, yield, rupture, stiffness, fatigue resistance, and ductile strength are all significantly affected by porosity, a microstructural feature [59,60]. The superior mechanical properties are often seen in scaffolds with low porosity [61]. In nanofibrous scaffolds, mechanical characteristics are typically reported to decrease as porosity and pore diameter increase [34,62]. The stiff porous nanofibers located in the nanofibrous webs with strongly packed structures and enhanced molecular orientation have high tensile modulus and strength and low elongation at breakage. Reduced porosity and smaller pore sizes also improve the ductility of the material [63]. On the other hand, larger pore sizes lead to massive surrounding fibrous tissue accumulation post-implantation, which significantly reduces compliance, whereas low porosity limits endothelialization, negatively impacting antithrombogenicity [64]. The graft’s flexibility is reduced due to the extensive fibrous accumulation caused by the large pore diameters, and high-porosity scaffolds are weaker than low-porosity ones. On the other hand, sufficient porosity (>80%) is usually necessary to simulate vascular distensibility. A detailed examination of burst strength is also essential to bring a promising graft through the stages of in vivo investigation and further clinical studies [65,66]. Interestingly, it has been demonstrated that the burst strength can decrease significantly after a certain porosity level because the low-porosity scaffolds are too fragile to withstand high pressures [67]. The more flexible high-porosity scaffold had a larger strain at rupture, burst pressure, and suture retention strength than the low-porosity scaffold with more closely packed fibers. When Young’s modulus of the two grafts was compared, the low porosity graft had higher maximum stress and was stiffer than the high porosity grafts. On the other hand, bilayered grafts having layers of both high and low porosity exhibited performance outcomes that were comparable to those of monolayer grafts. Therefore, despite the use of the same polymer, different microarchitectures may provide mechanical properties that are noticeably different [68]. Additionally, the in vitro and in vivo mechanical performances of the grafts should be considered.

In addition to the porosity, pore size, and inner connectivity of pores, static permeability is another critical parameter that influences the penetration and proliferation of cells as vascular graft performance. The permeability affects the molecular exchange between the enclosed graft and the surrounding blood environment. Permeability depends on the electrospun scaffolds’ packing density, porosity, and pore size. Densely packed fibers result in poor porosity and permeability, which hinder cellular infiltration inside the scaffolds, thus limiting the penetration distance of cells. In these circumstances, the oxygen and nutrient diffusion is limited, and cells can survive only on the surface. A perfectly permeable vascular graft should prevent immunogenic molecules from entering and permit the free transportation of oxygen, essential nutrients, and metabolic waste of cells [69]. To maintain a cell’s expected growth, the permeability of TEVGs must be sufficient to transport oxygen and nutrients and export waste between the microenvironment of cells and the blood.

#### 5.1.2. Fiber Orientation

Recent studies on the electrospinning of aligned fibers mainly concentrate on the configuration of the collector system, such as parallel electrodes, metal rotating discs, and mandrels. The main concerns of researchers are the linear velocity of the collector surface and the effects of the collection settings on the electric field. It has been stated that the electrical properties of the solvent, along with the collector speed, have a significant impact on the level of fiber orientation [70]. It is challenging to achieve the high speeds greater than 10,000 min^−1^ required to obtain fiber orientation by using rotating mandrels with diameters less than 6 mm. As a result, large-diameter rotating collectors (630 mm, 100 mm, 32 mm, and 640 mm) were used in many studies to achieve high rotational speeds and eliminate the resonance frequency concern [71]. It has also been shown in the literature that the polymer type is another factor that affects the aligned fiber morphology. Some polymers can align crimp-like, whereas others are oriented in the flat form [72].

Fiber orientation has been regarded as one of the most important characteristics of scaffolds since it affects both cellular orientation and the mechanical characteristics of prostheses used as vascular grafts [73,74]. The main factor influencing cell development behavior is fiber orientation, and cells on scaffolds typically create a phenotypic morphology and grow effectively based on fiber alignment [75,76]. Furthermore, it has been demonstrated in studies that radially oriented fibers encourage SMC penetration and alignment [77].

On the other hand, there is a significant correlation between the radial elastic modulus of the tubular scaffolds and the direction of fiber orientation. Circumferentially aligned fibers provide higher radial elastic modulus, and the Poisson effect confirms the distribution of fiber orientations in terms of mechanical characteristics [78]. In contrast to their orientated counterparts, randomly distributed fibers significantly improve the suture retention strength (SRS). This result is unexpected as efficient scaffold designs are usually approached with orientation to enhance mechanical properties. Thus, a multilayer strategy for vascular substitutes with carefully selected fiber orientations is necessary to provide the ideal balance of compliance, burst pressure strength, and SRS, particularly at the anastomotic site [79]. Modifying fiber orientation enables the control of graft compliance [80]. Oriented fibers display better modulus, tensile strength, and burst strength values, as well as reduced compliance when strained in the direction of orientation, which is related to the stiff structure of the material [81].

#### 5.1.3. Wall Thickness

Along with the previously mentioned factors, wall thickness is a crucial factor in designing vascular grafts since it affects the biomechanical characteristics, compliance, burst pressure resistance, and biological activities. Native vessels are reported to have walls with thicknesses ranging between 400 and 1000 μm [82]. Increasing the electrospinning duration will result in larger walls for the vascular scaffolds, significantly enhancing their circumferential tensile strength and suture retention strength [83]. Suture movement is more challenging in grafts with thicker walls, which provide increased fiber overlapping and enhanced binding force. However, the increased wall thickness is unfavorable for graft porosity and compliance [84]. Vascular grafts with a thinner wall thickness are more permeable and have greater mass transfer than the ones with a greater wall thickness in vivo. Hence, they have better cell proliferation and attachment performance than grafts constructed with thick layers [85]. Additionally, studies have demonstrated that as wall thickness is increased, vascular graft compliance decreases [86]. Compliance mismatch among the synthetic vascular scaffold and the native blood vessel also causes a change in hemodynamics, which then affects wall shear stress (WSS) and creates irregular flow patterns. Thus, undesirable biological responses are triggered by inconsistent mechanical signals that result in intimal hyperplasia [87]. In several investigations, the wall thickness has been decreased to produce compliant grafts similar to the native vessels; nevertheless, this can lead to poor bursting strength, which might not be adequate for implantation. The thickness of the graft wall also has an impact on blood permeability and graft handling during surgical procedures. Hence, it may be challenging to achieve a proper balance between all mechanical and biological properties and design parameters, especially blood leakage, cell permeability, burst strength, and compliance, when deciding on the wall thickness of the vascular grafts [88]. This makes determining the ideal wall thickness for vascular grafts extremely important.

#### 5.1.4. Number of Layers

As previously mentioned, vascular tissue engineering aims to imitate the construction and activities of native vessels that are composed of three layers known as the *tunica intima*, *tunica media*, and *tunica adventitia*, which provide high strength, elasticity, and compliance as well as outstanding hemodynamic function and anti-thrombogenicity [89]. Different roles are accomplished by each layer within the blood vessels. For instance, the endothelium layer of a native blood vessel is a well-organized monolayer, and the alignment of endothelial cells can regulate biological signaling such as intracellular protein expression, cytoskeleton development, and cellular interactions, whereas the middle layer involves spindle-shaped and circumferentially oriented SMCs that significantly affect the elasticity, mechanical strength, and vasoactive reactivity of blood vessels [90]. The reported mechanical and biological incompatibility of monolayered electrospun vascular scaffolds has led to the development of vascular prostheses with multilayers as an alternative technique for mimicking the characteristics of these layers [91]. In this regard, the middle and outermost layers should have a higher porosity to encourage SMC migration, whereas the inner layer should have a lower porosity to promote EC proliferation and limit blood permeability [92]. According to the researchers, creating multi-layered vascular scaffolds that imitate the mechanical and structural features of the native vessel walls is a useful way to mimic the functions of the *media* and *intima* layers [90,93]. Additionally, fabricating synthetic vascular scaffolds consisting of multiple layers with unique mechanical characteristics enables achieving a particular J-shaped stress–strain curve as in native blood vessels that show non-linear stress–strain behavior that provides the vessel’s resilience and, as a result, helps prevent aneurysms [94]. Due to the integrated mechanical features of the layers, the composite effect has been observed in stress–strain graphs in the work by Yalcin Enis et al. [71] by creating bilayered scaffolds with layers of random and orientated fibers of PCL and PLC polymers with various molecular weights. Therefore, by optimizing the fiber diameter, fiber alignment, pore size, wall thickness, material type, or their combinations, multilayer designs should be created to meet the requirements of vascular grafts in separate layers.

### 5.2. Material Selection

Synthetic vascular grafts made of non-biodegradable materials, including ePTFE, Dacron, and PU, which are commercially utilized, are not suitable for manufacturing grafts with diameters smaller than 6 mm, which are required to replace the saphenous vein, internal mammary artery, or radial artery as a vascular substitute because of poor patency, compliance mismatch, thrombosis, and ineffective neo-vessel development [12,77,95]. The drawbacks of currently available materials have prompted scientists to design biodegradable synthetic vascular grafts to enhance native vessel regeneration and reconstitute a functional arterial composition. However, when employed in animal experiments, these grafts have shown severe failure because of aneurysms, intimal hyperplasia, and thrombosis. These outcomes are probably brought on by the regenerated grafts with an insufficient amount of elastin [96].

Multiple biopolymers, including synthetic and natural ones, can be used to construct vascular grafts from electrospun fibers [97]. These materials are utilized to create a vascular scaffold that is physiologically suitable, and they should be chosen based on the graft structure, desirable biodegradation rate, and capacity for cell adhesion [38]. The material and architecture of small-caliber TEVGs have a significant impact on their biocompatibility, non-toxicity, non-immunogenicity, mechanical properties, ease of handling, and storability [98]. Furthermore, the scaffolds need to support host tissue remodelling and tissue regeneration during biodegradation and withstand inherent biological stresses as in biological systems to resist long-term issues including infection, intimal hyperplasia, stenosis, calcification, and aneurysmal dilatation [99]. While a rapid degradation rate may improve regeneration efficiency, it may also diminish tissue performance and damage mechanical qualities. In contrast, a slow degradation rate may hinder the development of neo-tissues. Therefore, it is essential to maintain a balance between the rates of vascular regeneration and biodegradation [37]. Additionally, the significance of in vivo foreign body responses of monocytes and macrophages to biomaterials is crucial for developing neo-vessels and thrombosis; as a result, strategies for material choice and manufacturing that control macrophage phenotype have drawn considerable attention [100]. Since it is normally impossible for one material to satisfy all of these qualities, mixing several polymers to form a hybrid graft seems to be an effective way to fabricate TEVGs [8]. These composite scaffolds can be thought of as innovative smart biomaterials that have the potential to produce TEVGs since they blend the advantages of natural polymers, including biocompatibility and biochemical capabilities, with the benefits of synthetic polymers, consisting of high strength, modifiability, and processability [101]. Some of the most commonly used biopolymers in vascular tissue engineering applications are given in Table 1, with their advantages and disadvantages.

An ideal vascular graft should possess mechanical strength, compliance, suture retention strength, and a J-shaped mechanical response close to physiological levels. The mechanical responses of both passive (elastin and collagen fibers) and active components (SMCs) affect the mechanical reaction of the artery wall. When arteries are subjected to blood pressure, non-linear elastic behavior is seen as a J-shaped curve [131]. Many biomaterials and biological tissues have what is known as a J-shaped strain–stress curve, which illustrates how small increases in stress initially lead to enormous elongation, but as the material stretches further, it stiffens and becomes more difficult to stretch [132]. Elastin fibers are mainly responsible for the compliance of vessel walls at low pressures, whereas high stiffness is mainly caused by the mechanical reaction of collagen at high pressures. As pressure increases, collagen fibers start aligning and orienting, lowering arterial compliance. Therefore, utilizing collagen and elastin and mimicking their crimpy architecture appears to be a suitable method for creating a small-caliber vascular graft that simulates this mechanical reaction [131]. In order to provide sufficient compliance and structural integrity of TEVGs, the co-spinning of both elastin and collagen has been used as a strategy to imitate the artery’s three-layered architecture [133].

A natural contractile-like SMC phenotype can be differentiated to resemble the composition of native blood vessels by using ECM proteins such as collagen type I and insoluble elastin, which have superior viscoelastic capabilities. In addition to their outstanding biological features, natural polymers can be easily modified in terms of their mechanical characteristics and biodegradation rates through the change in their degree of crosslinking [134]. The polymers derived from ECM components collagen, elastin, fibrin, and gelatin are utilized to create TEVGs. There are many studies targeting the improvement of the integrity of collagen-based grafts to overcome the inadequate mechanical qualities, including anastomosis strength, burst pressure, and tensile strength. Elastin and gelatin grafts have similar behavior. In addition, dynamic culture has enhanced the mechanical behavior of fibrin grafts developed from in vitro-produced fibroblasts, providing compliance close to natural blood vessels [8]. The J-shaped mechanical behavior is generally achieved when natural biopolymers such as collagen, elastin, or fibrin are used.

However, natural polymers’ properties differ from sample to sample, and it might be challenging to find consistently suitable production conditions. They are also weak, which makes it tricky to withstand intense physiological forces. Although synthetic biopolymers with mechanical properties similar to collagen and elastin appear to be promising materials for replacement, using them in a neat form causes a decrease in compliance as stress increases. This makes achieving J-shaped reactions in vascular grafts difficult [131]. Some of the most studied synthetic biopolymers for vascular scaffolds are biodegradable polyesters, including PGA, PLA, PLLA, their copolymer PLGA, and PCL [10]. When compared to natural polymers, synthetic polymers have several advantages. First, they are simple to produce due to their physical and chemical characteristics. Despite their ability to help restore damaged tissue structure and activity, these biomaterials have limited cell attachment locations and thus need chemical modifications. The tensile strength, Young’s modulus, and degradation rate can be predicted and repeated over a wide range. These polymers vary in their degree of biodegradability, biocompatibility, and mechanical characteristics, but no single polymer provides the ideal mix of all of these crucial characteristics [135]. Therefore, utilizing various polymers to construct hybrid grafts that may offer ideal features similar to native vessels is considered a promising technique.

Tissue engineers have been able to adjust TEVG features thanks to various polymers and production methods, but choosing the ideal mix of graft properties is still difficult to accomplish [136]. Therefore, the design and material selection should be considered together rather than individually, as the scaffold’s characteristics depend on its morphology and material.

## 6. Mechanical Forces Acting on the Vascular Grafts

Sufficient mechanical strength and Young’s modulus in the longitudinal and radial directions are essential for clinical trials of vascular grafts because these scaffolds must withstand repeated mechanical stresses, including expansion, shrinkage, bending, and stretching under the in vivo conditions affected by blood flow and body movements [137]. In creating vascular grafts, it has been challenging to balance adequate mechanical strength to endure physiological pressures and compliance similar to native vessels to avoid unfavorable hemodynamic fluctuations [31]. In order to imitate in vivo conditions, vascular tissue engineering needs a platform that mimics the hemodynamic shear and regular forces that vascular tissues experience in the body in the radial, circumferential, and longitudinal directions. Shear stresses are tangential frictional forces directly affecting ECs and are delivered to SMCs by interstitial flow and signaling [138]. Numerous hemodynamic forces, including flow shear stress, frictional forces parallel to the vascular wall produced by blood flow, and circumferential stress perpendicular to the vascular wall brought on by transmural pressure, affect all blood vessels. Vascular SMCs and ECs are likewise affected by these physical stresses; variations in stress can trigger intracellular signaling pathways that affect the cellular activity and blood vessel formation [139]. The endothelium performs the role of a mechanoreceptor, sensing variations in blood flow, shear stress, and pressure and then causing the secretion of signaling molecules that cause the SMCs to dilate or constrict. Pulsatile flow and mechanical stresses in the blood vessel lumen also have an impact on patency levels. Laminar shear stress is essential for maintaining the required endothelium morphology [38]. The pulsatile blood pressure periodically exposes the vascular wall to cyclic circumferential stresses of about 100–150 kPa, causing average strains of 10–15%. Further, on the vascular wall of humans, blood flow produces an oscillatory shear stress of 1–5 Pa, which differs based on body size and vessel type. Even though shear stresses are five times less intense than circumferential stresses, they still have a considerable effect on cellular activity [140].

**Figure 3:**
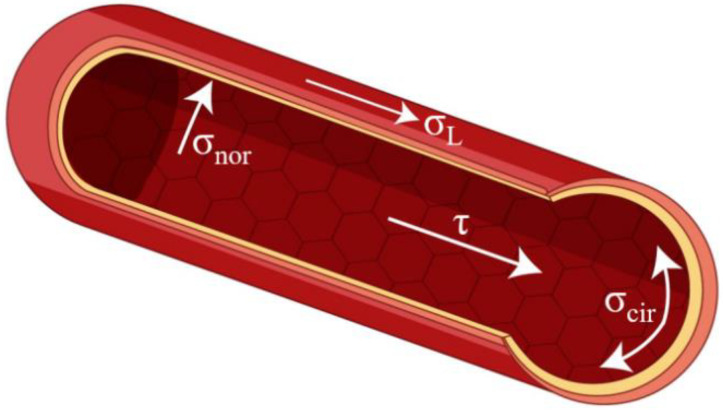
The mechanical stresses on blood vessels.

Therefore, it can be noted that vascular grafts are exposed to four hemodynamic stresses: shear stress (τ, tangential frictional forces acting on ECs attributable to blood flow); luminal pressure (σ_nor_, a cyclic normal force due to blood pressure); cyclic circumferential stress (σ_cir_, a circumferential mechanical stretch due to blood pressure); and longitudinal stress (σ_L_,) [141], which are illustrated in Figure 3. Thus, blood pressure produced by pulsatile blood flow also moves perpendicular to the EC matrix [142].

### 6.1. Shear Stress

The tangential element of frictional forces brought on by the blood flow in a lumen is known as shear stress. The shear stress unit in the SI system is the Pascal (Pa). The cardiovascular system frequently makes use of dyn/cm^2^ (1 Pa = 10 dyn/cm^2^) [143]. Shear stress (*τ*), which is parallel to the vessel wall, is used to describe the frictional force acting on the vascular endothelium. ECs face shear stress that ranges from 1 to 6 dynes/cm^2^ in the circulatory system and from 10 to 70 dynes/cm^2^ in the arteries, with an average of 20 dynes/cm^2^ [144]. When blood shows laminar flow, shear stress is calculated as

τ = 4*μ*Q/(πr^3^) (1)

where *μ* is the viscosity, Q is the flow rate, and r is the radius of the vessel. The shear stress in arteries with large diameters typically ranges between 5 and 20 dyn/cm^2^, although under conditions of high systolic pressures, significant instant values can reach 40 dyn/cm^2^ [145].

As a result of blood flow, shear stress triggers ECs to convert mechanical sensations into intracellular signals that change cellular processes such as proliferation, apoptosis, infiltration, permeability, and regeneration as well as gene expression. These changes are crucial for maintaining the homeostasis of vascular system and the mechanisms underlying blood flow-induced circumstances such as angiogenesis, vascular regeneration, and atherogenesis [146,147]. Additionally, endothelial progenitor cells (EPCs) grown from human peripheral blood elongate and align longitudinally towards the flow direction when subjected to laminar shear stress [147]. Vasodilation and vasoconstriction triggered by flow are affected by WSS. Intimal hyperplasia is the outcome of alterations in the flow pattern that are brought on by both high and low shear stresses. Stress concentration in anastomotic regions and compliance mismatch between the native vessel and the scaffold are the two factors that contribute to abnormal WSS. Therefore, it is critical to avoid compliance mismatch in order to reduce these disruptive flow patterns [64]. Shear stress can also lead weakly adhered ECs to detach from the lumen, resulting in thrombosis in vascular grafts lacking endothelial lining when blood encounters a surface other than the endothelium. In numerous investigations, exposing vascular grafts to shear forces in pre-implantation improved EC adhesion [148].

### 6.2. Luminal Pressure

Pulsatile blood flow creates a tensile stress as a result of the normal force that is perpendicular to the vessel wall and results in cyclic strain [149]. ECs can sense and respond to normal stresses. Cell adaptation systems can be inappropriate and cause disease and significant changes in the cell phenotype when they are subjected to extreme conditions such as mechanical stress from continuous and high-intensity stretching [150].

### 6.3. Cyclic Circumferential Stress

This is the periodic stretching of arterial wall components that is produced by periodic increments in transmural pressure difference and normal forces caused by blood flow [143]. Axial and circumferential strains have a significant impact on EC morphology, vascular cell proliferation, and matrix remodeling [151]. Endothelial cells adjust their phenotype and active signaling mechanisms in response to cyclic circumferential strain by orienting themselves perpendicular to the force vector [152]. Mechanical stretch, which is detected by mechanoreceptors, also controls how the SMC responds. Physiological pulsatile circumferential stress on the arterial wall enables SMCs to show contractile response. Circumferential stress consequently influences gene expression as well as SMC processes such as proliferation, survival/apoptosis, diffusion, and ECM remodeling. Circumferential stress varies from 1 to 2 × 10^6^ dynes/cm^2^, based on the anatomical region [151].

Circumferential stress is studied more extensively as it provides data on the dimensions of the vascular luminal diameter and wall thickness changes based on blood pressure variations. It is possible to determine circumferential stress (σ_cir_), which is frequently represented in dynes/cm^2^, as follows:

σ_cir_ = (P.r_i_)/t (2)

where P is the internal pressure, r_i_ is the inner radius, and t is the wall thickness of the graft [153,154]. The mean circumferential stress is considered to better respond to slight variations in the *intima* thickness and increases with larger wall thicknesses [155,156]. The internal pressure also can be calculated as follows:

P = F/(2r_i_L) (3)

where F is the force when it is exposed to P, and L is the length in the z direction [157]. In addition, the circumferential strain measures the change in the internal diameter brought on by a change in the intraluminal pressure. The following formula can be used to determine strain (ε_i_), which has no units:

εi = (D_i_ − D_0_)/D_0_ (4)

where D_i_ is the diameter at a particular pressure, and D_0_ is the reference diameter [153]. Under physiological blood pressure of 100 mmHg, the mean circumferential stress of the coronary arteries is around 150 kPa, whereas the strain is approximately 10–15% [158].

### 6.4. Longitudinal Stress

All arteries experience longitudinal (axial) stresses varying from 40% to 65% in vivo [159]. Physiological alterations can alter these longitudinal stresses because arterial tethering exposed by surrounding tissues maintains them. Key mechanical signals that encourage arterial remodeling are also provided by longitudinal stresses, such as changes in shear stress and circumferential strain [160]. A longitudinal strain also promotes cell proliferation in the artery wall while preserving arterial wall function [161]. The distending force in the longitudinal direction results in internal longitudinal stress (σ_L-P_). On the other hand, a second tensile force creating longitudinal stress in the latter direction exists due to the arterial tethering (σ_L-T_) caused by surrounding tissue along its length. The formulas given below can be used to calculate the internal longitudinal stress (σ_L-P_), the longitudinal stress caused by arterial tethering (σ_L-T_), and total longitudinal stress (σ_L_):

(5) $$\sigma_{L-P}=\frac{Pr_{i}}{2t}$$

σ_L-T_ = F_L-T_/(π (r_e_^2^ − r_i_^2^) (6)

**Figure 4:**
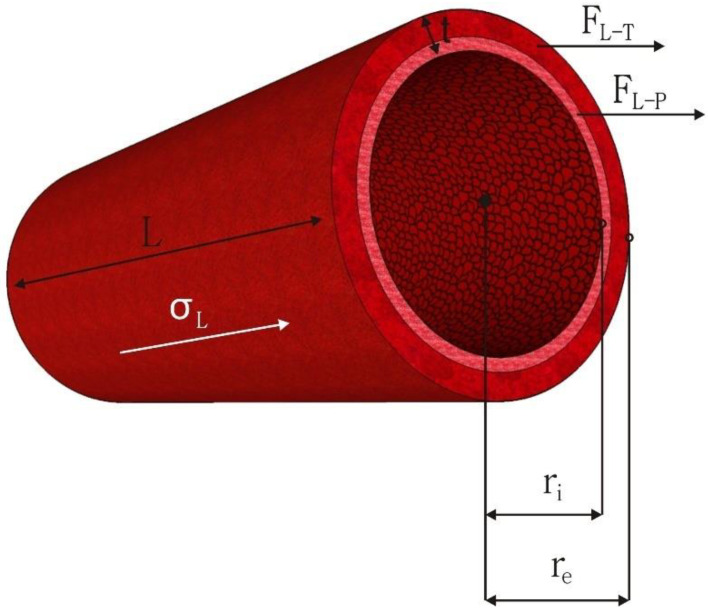
The dimensional variables of a vascular graft and longitudinal force components.

σ_L =_ σ_L-P_ + σ_L-T_ (7)

where the variables are the P, r_i_, external radius (r_e_), t, and the longitudinal forces comprising the forces resulting from the blood pressure (F_L-P_) and tethering (F_L-T_) in Equations (5) and (6). The total longitudinal stress (σ_L_) equals the sum of stress due to pressure (σ_L-P_) and stress due to tethering (σ_L-T_) given in Equation (7) [19]. All dimensional variables and the force components used in these equations are represented based on the vascular graft in Figure 4.

The human femoral artery has a longitudinal elastic modulus of 978 kPa and longitudinal maximum stress of 65 kPa, placing it at the maximum limit of small-diameter vascular grafts [162]. Additionally, it has been discovered that when the artery is stretched longitudinally by 48% of its load-free length in vivo, the longitudinal stresses are greater than the circumferential stresses [163].

Therefore, the physical and mechanical properties of vascular grafts, such as dimensions, compliance, bursting strength, elasticity, and Young’s modulus, must be properly provided by considering the design criteria that have a major impact on their long-term performance in order for them to resist all of the aforementioned forces.

## 7. Mechanical Characteristics of Vascular Grafts

Recent methods for enhancing the clinical efficacy of vascular grafts and preventing graft failure brought on by intimal hyperplasia, thrombosis, aneurysm, blood leakage, and occlusion have been documented in the literature. These methods include creating grafts with adequate compliance, elasticity, ultimate tensile strength, ultimate strain, Young’s modulus, burst pressure, and suture retention strength to withstand all of the physiological stresses that native vessels experience [81,164,165,166,167].

According to recent studies, it is essential to develop vascular prostheses that have sufficient tensile strength and strain and also exhibit a nonlinear, J-shaped stress–strain behavior similar to human coronary arteries [101]. The stress–strain curve shows that blood vessels have nonlinear J-shaped mechanical behavior, with an initial elastic region at low strain due to the presence of elastin, followed by an increase in stiffness with a curved transition at high strain due to the presence of collagen. Collagen and elastin, two structural proteins, work together synergistically to produce the J-shaped curve observed in strained materials. This mechanical response provides adaptation and protection to aneurysm generation, which is identified as a localized expansion or ballooning of a section of an artery higher than 50% of its normal diameter, primarily induced by a weakening of the vascular wall exposed to primary stress [168,169]. Unfortunately, the J-shaped mechanical behavior of native vessels has not been replicated in most trials fabricating vascular prostheses to substitute coronary arteries. Some techniques have been used to solve these issues, such as constructing the middle and outer layers to approximate the structural configurations of collagen and elastin fibers found in native blood vessels [170].

Native blood vessels are dynamic, flexible, and strong tissues that can withstand physiological stresses. The constructed vessel must resist pressure and pulsatile blood flow without rupturing or membrane leakage. Burst pressure is, therefore, among the most crucial elements in assessing whether a material is suitable for implantation and describes the amount of pressure the scaffolds can endure before failing [171]. Generally, TEVGs designed for replacement are expected to have burst pressures greater than 2000 mmHg [172]. In addition, TEVGs must be able to withstand distortion and compression and show adequate tensile and shear strength to guarantee that the scaffold can resist the tensile stress caused by suturing during implantation, to prevent rupture, scattering, wear of the edges, and tearing of the seams, and to sustain circumferential strength to withstand hemodynamic forces [84]. The suture retention strength analysis is employed to calculate the magnitude of force needed to rupture the scaffold wall or rip a suture from a structure. The suture retention strength of the human saphenous vein is 1.81 N [173].

**Figure 5:**
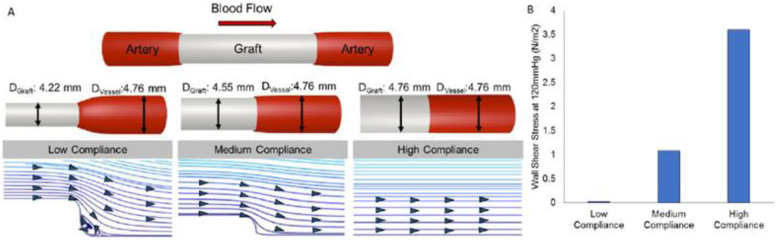
The effect of compliance on blood flow profiles and WSS [174].

Compliance, also known as the inverse of stiffness or the change in vessel diameter over time as a function of pressure, is a term used in vascular tissue engineering to describe the ability of the vascular scaffold to stretch circumferentially as a reaction to pulsatile pressure [87,88,174]. The performance of the prosthesis is affected by compliance mismatch between a native artery and a vascular replacement, which results from different mechanical behaviors that cause a mismatch in diameter change and can reduce patency due to intimal hyperplasia [87,175]. Low WSS is considered to be the outcome of the compliance mismatch interrupting the flow at the distal anastomosis. Because of the low WSS, the vessel wall tries to restore the flow disruption by thickening the *intima*, which finally causes the scaffold to occlude [174]. Additionally, a longer residence duration of atherogenic particles, associated with the low WSS, may trigger blood particle accumulation or adherence to the artery wall, encouraging plaque growth and intimal thickening. Thus, lower WSS may be negatively impacted by arterial compliance mismatch, which could lead to particle deposition in stiffer vascular wall regions and increase the risk of arterial disease [176]. In many studies, a compliance mismatch results from the stiffness of commercially available synthetic polymers, such as ePTFE and Dacron [177]. Moreover, it is well reported that intimal hyperplasia, anastomotic aneurysms, or pseudoaneurysms can result from excessive stress at the suture locations caused by a compliance mismatch between the native vessel and the Dacron scaffolds [178]. Therefore, avoiding these issues and obtaining effective graft performance depend on developing a scaffold with compliance properties close to native vessels. Figure 5 illustrates how the compliance level affects blood flow patterns and WSS.

## 8. Current Studies Guiding the Literature

Some studies focusing on the effect of design criteria on both cellular activities and mechanical properties and shaping the literature with seminal findings are discussed below.

Fiber diameter and pore geometry, which vary accordingly, are among the design parameters that should be prioritized in determining mechanical properties. Matsuzaki et al. [179] studied the influence of pore size on neoarterial tissue regeneration and graft stability over the regeneration phase using electrospun scaffolds. Scaffolds with an inner diameter of 5 mm were constructed using electrospun PCL fibers with four distinct porosity and pore sizes in the outermost layer (79% (4 μm), 82% (7 μm), 83% (10 μm), and 85% (15 μm) and heparin-conjugated PLCL sponge as the inner layer. All of the grafts were implanted in adult female sheep. Only scaffolds with pore sizes of 4 μm were demonstrated to resist dilatation for up to a year. Grafts with wider pore sizes improved cell infiltration, but neotissue could not regenerate quickly enough to provide the mechanical strength required to resist dilatation. Grafts with pore sizes of 10 and 15 μm had lower strength than that of the sheep carotid artery, which was approximately 3 MPa, but grafts with pore sizes of 4 and 7 μm had higher strength, around 4 MPa. Furthermore, the highest level of compliance was attained with the 10 μm pore-sized graft at roughly 1% mmHg^−1^, which was still lower than that of the sheep carotid artery. It is also possible to examine the effect of pore size on mechanical properties through fiber diameter. Wang et al. [51] used electrospinning to manufacture PCL vascular grafts with an inner diameter of 2 mm, and thicker fibers were generated to achieve a structure with macroporosities to improve the scaffold’s cell infiltration capabilities. While the fiber and pore diameters of these macroporous scaffolds were around 5–6 μm and 40 μm, respectively, these values were around 0.7 μm and 5 μm, respectively, in microporous scaffolds. The strain value of grafts with thicker fibers was approximately 639.20% higher than the strain value of grafts with thinner fibers, which was 168.40%, indicating that thicker-fiber grafts were substantially more rigid than thinner-fiber grafts. Grafts with thinner and thicker fibers had Young’s moduli of 17.44 MPa and 21.00 MPa, respectively, indicating that an increase in fiber diameter resulted in a slight rise in Young’s modulus. The ultimate tensile stress value of a thinner fiber graft was 13.35 MPa, but the ultimate tensile stress of thicker fiber grafts was 8.72 MPa, suggesting that increasing fiber diameter generated decreased tensile stress. Similarly, Valence et al. [56] used electrospinning to create PCL bilayered grafts with a 2 mm inner diameter, integrating a high-porosity layer with a low-porosity layer on either the luminal or adventitial side. There were four types of grafts produced: no barrier (high-porosity scaffold), inside barrier (low-porosity inner layer and high-porosity outer layer), outside barrier (high-porosity inner layer and low-porosity outer layer), and only a barrier (low-porosity scaffold). The results show that high-porosity grafts had a greater strain at break, burst pressure, and suture retention strength than low-porosity scaffolds. The maximum stress (6.09 MPa) and Young’s modulus (12.0 MPa) of the low-porosity scaffold were greater than those of the high-porosity scaffold, at 4.98 MPa and 6.09 MPa, respectively. The only-barrier scaffold was more rigid than the no-barrier, inside-barrier, and outside-barrier grafts. On the other hand, the burst pressure and suture retention strength was higher for the more compliant no-barrier graft than for the only-barrier graft. Thus, it was emphasized that, despite using the same material, different microarchitectures could result in significantly different mechanical properties.

The wall thickness of vascular scaffolds, on the other hand, is another structural parameter that directly affects mechanical features, including compliance, burst pressure, suture retention, and tensile strength. Johnson et al. [31] investigated the effect of polymer choice and wall thickness on the mechanical properties of a vascular prosthesis. First, they evaluated the biomechanical properties of electrospun vascular grafts made of various biopolymers such as PCL, chitosan, PLCL, and PLLA, with a diameter of 6 mm and a wall thickness of 650 μm. The burst strength of the scaffold made of PLGA was 3.3 MPa, while the burst strength of the scaffold built of PCL was 0.8 MPa. The burst strengths of the human carotid artery, human saphenous vein, and ePTFE graft were all much lower than those of the electrospun scaffolds, ranging between 0.1 and 0.5 MPa. Among all, the PLLA graft exhibited the highest suture retention strength value of 1022 g. The best compliance value was 8.2% mmHg^−1^ in PLCL grafts, while PLLA had a compliance value of approximately 3.8% mmHg^−1^, close to that of the human coronary artery. The ePTFE graft had the lowest compliance value of 1.6% mmHg^−1^. They also electrospun PCL grafts with varying wall thicknesses and studied their mechanical properties to see if sidewall thickness affects scaffold compliance and burst pressure. For wall thicknesses ranging from 400 to 1000 µm, compliance remained constant at 2–4% mmHg^−1^. On the other hand, PCL grafts exhibited significant variance in compliance values, ranging from 2 to 11% mmHg^−1^ at wall thicknesses of less than 400 µm. The burst pressure increased as the graft sidewall thickness increased. For PCL grafts with wall thicknesses ranging from 200 to 1000 µm, the burst pressure values increased linearly from 0.6 to 2.9 MPa. As a result, it was underlined that the wall thickness has a significant and more robust effect on the mechanical properties. The wall thickness can be adjusted by increasing or decreasing the electrospinning time. Therefore, optimizing this time to see the wall thickness effect is an important research topic. The goal of Bazgir et al. [180] was to evaluate the properties of PLGA and PCL-based nanofibrous scaffolds, as well as the effect of degradation on their structural properties. Six scaffolds were built by electrospinning, three with PCL and the others with PLGA, with processing times of 30, 60, and 90 min. It was observed that there was a clear relationship between the duration of the electrospinning and the tensile strength. In the case of PCL scaffolds, the ultimate stress and strain values were 0.99 MPa and 24.03% for the scaffolds fabricated in 30 min and 1.49 MPa and 28.15% for the scaffolds produced in 90 min, respectively, whereas 1.03 MPa and 34.36% for PLGA scaffolds manufactured in 30 min and 1.76 MPa and 36.33% for PLGA scaffolds produced in 90 min. Thus, both types of scaffolds demonstrated more robust mechanical properties with longer spinning periods, resulting in higher wall thicknesses. When the tensile strength and elongation values of PCL and PLGA scaffolds with a 90-min processing time were compared, the PLGA membrane was demonstrated to be more elastic and durable than the PCL membrane. The research reveals a relation between the electrospinning processing time and tensile strength. The scaffold became thicker and stronger when the electrospinning duration was increased.

Aside from the aforementioned criteria, several studies have focused on the effect of fiber orientation on the mechanical properties of vascular grafts. Many researchers claim that, based on the architecture of the native artery, fiber orientation can aid in improving mechanical properties, particularly tensile strength and burst pressure. Yalcin et al. [71] generated vascular scaffolds with a 6 mm diameter and a wall thickness ranging between 200 and 300 µm made of randomly distributed or radially oriented PCL and PLCL microfibers, and the effect of fiber orientation, polymer type, and the number of layers on the mechanical properties of the prosthesis was investigated. Bilayered grafts were created by combining layers with randomly distributed fibers in the inner layer and radially orientated fibers in the outer layer. In general, the fiber orientation in the radial direction utilized in the outer layers contributed to the burst and tensile strengths of the samples in the same direction. Among the single-layer grafts, the PCL grafts with oriented fibers had the highest ultimate tensile strength at 6.7 MPa in the radial direction and the lowest elongation at break, which was 80%. Oriented PLC samples, on the other hand, displayed elongation values greater than 900% due to their inherent high elasticity. In the case of bilayered samples, all of the samples had higher tensile strength and lower elongation values in the radial direction rather than the axial direction, owing to a greater number of fibers in that direction to bear stress but also fewer crossing points. Due to their extraordinarily elastic nature, both randomly distributed and radially orientated PLCL samples swelled at a lower pressure of around 562 mmHg but had the highest bursting resistance at 1500 mmHg. The findings supported the significant influence of polymer and graft construction on the mechanical behavior of scaffolds. Similarly, Grasl et al. [94] used an adjustable electrostatic field to alter the direction of the electrospinning jet, resulting in 2-mm-caliber vascular scaffolds containing circumferentially, axially, fenestrated, and randomly distributed PU and PLLA fibers. The influence of polymers and fiber orientations on the mechanical behavior of the prosthesis was evaluated and compared to that of the native rat aorta. The ultimate tensile force of PU and PLLA grafts composed of randomly oriented fibers was lower than that of the other fiber orientations. The grafts with circumferentially oriented fibers, on the other hand, had at least six times higher ultimate tensile forces than the grafts with randomly aligned fibers. Furthermore, the PU and PLLA prosthesis consisting of randomly oriented fibers showed a significantly lower maximum tensile force than the rat aorta (1.1 N) while the samples with circumferentially oriented fibers had much higher tensile forces than the rat aorta. Scaffolds built of both materials with randomly oriented fibers exhibited considerably lower burst pressures than other fiber orientations. Each fiber orientation contributed to bursting strength, close to or greater than the rat aorta’s burst pressure (1043 mmHg). All directions of the alignment resulted in a decrease in compliance. The PU scaffolds with randomly oriented fibers displayed the maximum compliance of 29.7% 100 mmHg^−1^, which is the closest value to the compliance of rat aortas (37.2% 100 mmHg^−1^). Because of their rigidity, PLLA scaffolds have a lower compliance value. None of the grafts with alternative fiber alignments or polymers could replicate the J-shaped mechanical behavior of the rat aorta, supporting the researchers’ view that multi-layered vascular scaffolds should be constructed to imitate the structure of the native blood vessel.

Polymers in mixed forms have been thought to be far more promising in tissue engineering applications than utilizing them alone. All polymers have unique properties that contribute to the various characteristics of the scaffolds. Finding the appropriate blend ratio is critical for attaining these scaffolds’ required and optimum properties. Gao et al. [37] investigated the influence of polymer ratios on the mechanical properties and degradability of 2-mm-caliber electrospun vascular grafts made of PCL and PLGA in several blending ratios, including 95/5, 90/10, 80/20, and 60/40. When the findings were analyzed, it was revealed that the mechanical properties of PCL/PLGA (95/5) and PCL/PLGA (90/10) were adequate for use as vascular prostheses because they did not exhibit ultimate tensile stress, strain, or bursting strength values under the range of original grafts. The stress-strain graphs indicated that the strength and elongation of blended samples decreased as the proportion of PLGA increased. Furthermore, increasing the amount of PLGA reduced the suture strength and burst pressure of the PCL/PLGA blended scaffolds. The burst strength of the PCL/PLGA (95/5) and PCL/PLGA (90/10) scaffolds was found to be greater than 1500 mmHg, allowing them to be employed as a replacement for the native vessel. In another study examining the blend ratio effect on mechanical properties, Yang et al. [181] used electrospinning technology to create hybrid grafts of PCL and fibrin with blend ratios of 0/100, 10/90, 20/80, and 30/70. The results show that adding PCL to fibrin scaffolds significantly enhanced their mechanical properties. The burst pressure increased from 1347.43 to 1811.6 mmHg as the PCL ratio rose from 10% to 30%. On the other hand, the scaffold’s Young’s modulus dropped when the PCL content increased in all of the scaffolds. Finally, the PCL/fibrin (20/80) scaffold demonstrated balanced mechanical and degradability properties and high cell compatibility, indicating that it might be used as a tissue engineering platform for vascular grafts. However, the blend effect may not always combine the best result of both polymers as desired. Bolbasov et al. [182] used an electrospinning method to manufacture scaffolds comprised of PCL, PLLA, blended PCL/PLLA, and PLLA/PCL copolymer (PLC7015), emphasizing the relevance of material selection. Due to their highly crystalline structure, PLLA nanofibrous scaffolds showed the highest tensile strength (13.2 MPa) and lowest elongation (95%) values with uniaxial stretching. PCL and PLC7015 nanofibrous scaffolds’ semicrystalline structures resulted in moderate strength values of 8.2 MPa and 9.1 MPa, respectively. PLC7015 has a lower crystalline structure than PCL, resulting in a maximum elongation value of 560%. According to the researchers, the PCL/PLLA exhibited the lowest strength at 4.7 MPa due to thermodynamic incompatibility at the molecular scale. The PCL/PLLA graft differed significantly from the PLC7015 graft in strength and elongation. Thus, when establishing design parameters, the effect of polymers and the method used to combine different types of polymers (either copolymerization or blending) on the properties of the scaffolds should be considered.

## 9. Conclusions

In this review, in-depth research has been conducted by firstly giving the fundamentals of the structure of the blood vessels, the requirements for the ideal vascular prosthesis, and the electrospinning method for fabricating vascular membranes. The significance of the correct determination of the design parameters to achieve the most ideal vascular grafts was then emphasized by giving their impact on material function, particularly from the standpoint of the mechanical and biological performance of the scaffolds. In order to have a better understanding of the importance of the mechanical properties of the vascular graft that can be used as a replacement to maintain the damaged tissue functions, the physiological forces acting on blood vessels and their consequences on physical and biological activities are also explained in detail. In the final stage, the theoretical part of the review is supported by the examination and comparison of the recent experimental findings in the literature. This study aims to guide researchers working in this field by providing the literature with a comprehensive and comparative summary of the most recent studies.

## Figures and Tables

**Table 1:** Some of the advantages and disadvantages of natural and synthetic biopolymers used in tissue engineering.

Type	Biopolymers	Advantages	Disadvantages	References
Natural	Collagen	Supports EC and SMC attachment Biocompatible	Shows thrombogenicity Lack of mechanical strength	[102,103,104]
	Alginate	Biocompatible Gel forming ability Non-toxic Biodegradable Easy to process	Low stability Poor mechanical and barrier characteristics Requirement of combination with other biopolymers	[105,106,107]
	Chitosan	Formability Fabricability with other biopolymers Anticoagulant ability Biocompatible Biodegradable	Poor mechanical strength Low stability Low spinnability in electrospinning Toxicity	[108,109,110]
	Elastin	Mechanically flexible Resisting physiological pressures	Strong tendency to calcify Hard purification process	[111,112,113]
	Fibrin	Biocompatible Simple extraction procedure from patient’s blood Encourages cell adhesion and collagen synthesis Nonlinear elasticity Contributing compliance Resisting intense deformations	Low mechanical strength Fast degradation rate	[114,115,116,117]
	Gelatin	Low cost Biocompatible Biodegradable Low antigenicity No denaturation during electrospinning	Dissolution and loss of gelatin matrices under physiological conditions	[118,119]
Synthetic	PCL	Flexibility Structural stability Thermoplasticity Biocompatible Slow biodegradation	Slow biodegradation High hydrophobicity resulting in low cell affinity Low bioactivity Low oxygen permeability Low surface energy	[120,121,122,123]
	PLA	High tensile strength Non-toxicity Opportunity to control the polymer crystallization, shape, and hydrolysis Biodegradable Biocompatible	Acidic degradation byproducts Brittleness Poor wettability	[124,125,126,127]
	PGA	Fast degradation rate Good mechanical characteristics Biodegradable Biocompatible	Triggering inflammatory reaction Fast biodegradation rate resulting in a loss of mechanical performance	[128,129,130]

## Data Availability

Not applicable.

## References

[1] Ball S., Banerjee A., Berry C., Boyle J.R., Bray B., Bradlow W., Chaudhry A., Crawley R., Danesh J., Denniston A. (2020). Monitoring Indirect Impact of COVID-19 Pandemic on Services for Cardiovascular Diseases in the UK. Heart.

[2] Cardiovascular Diseases (CVDs). https://www.who.int/news-room/fact-sheets/detail/cardiovascular-diseases-(cvds).

[3] Kivimäki M., Steptoe A. (2018). Effects of Stress on the Development and Progression of Cardiovascular Disease. Nat. Rev. Cardiol..

[4] Krist A.H., Davidson K.W., Mangione C.M., Barry M.J., Cabana M., Caughey A.B., Donahue K., Doubeni C.A., Epling J.W., US Preventive Services Task Force (2020). US Preventive Services Task Force Behavioral Counseling Interventions to Promote a Healthy Diet and Physical Activity for Cardiovascular Disease Prevention in Adults with Cardiovascular Risk Factors: US Preventive Services Task Force Recommendation Statement. JAMA.

[5] Roth G.A., Mensah G.A., Johnson C.O., Addolorato G., Ammirati E., Baddour L.M., Barengo N.C., Beaton A.Z., Benjamin E.J., Benziger C.P. (2020). Global Burden of Cardiovascular Diseases and Risk Factors, 1990–2019: Update From the GBD 2019 Study. J. Am. Coll. Cardiol..

[6] Louridi N., Amar M., Ouahidi B.E. Identification of Cardiovascular Diseases Using Machine Learning. Proceedings of the 2019 7th Mediterranean Congress of Telecommunications (CMT).

[7] Melly L., Torregrossa G., Lee T., Jansens J.-L., Puskas J.D. (2018). Fifty Years of Coronary Artery Bypass Grafting. J. Thorac. Dis..

[8] Yuan H., Chen C., Liu Y., Lu T., Wu Z. (2020). Strategies in Cell-Free Tissue-Engineered Vascular Grafts. J. Biomed. Mater. Res. Part A.

[9] Kabirian F., Ditkowski B., Zamanian A., Heying R., Mozafari M. (2018). An Innovative Approach towards 3D-Printed Scaffolds for the next Generation of Tissue-Engineered Vascular Grafts. Mater. Today Proc..

[10] Carrabba M., Madeddu P. (2018). Current Strategies for the Manufacture of Small Size Tissue Engineering Vascular Grafts. Front. Bioeng. Biotechnol..

[11] Teebken O.E., Haverich A. (2002). Tissue Engineering of Small Diameter Vascular Grafts. Eur. J. Vasc. Endovasc. Surg..

[12] Hiob M.A., She S., Muiznieks L.D., Weiss A.S. (2017). Biomaterials and Modifications in the Development of Small-Diameter Vascular Grafts. ACS Biomater. Sci. Eng..

[13] Jouda H., Larrea Murillo L.L., Wang T. (2022). Current Progress in Vascular Engineering and Its Clinical Applications. Cells.

[14] Shakeel A., Corridon P.R. (2022). Mitigating Challenges and Expanding the Future of Vascular Tissue Engineering—Are We There Yet?.

[15] Leal B.B.J., Wakabayashi N., Oyama K., Kamiya H., Braghirolli D.I., Pranke P. (2021). Vascular Tissue Engineering: Polymers and Methodologies for Small Caliber Vascular Grafts. Front. Cardiovasc. Med..

[16] Townsley M.I. (2012). Structure and Composition of Pulmonary Arteries, Capillaries, and Veins. Compr. Physiol..

[17] Shrestha B., Prasai P.K., Kaskas A.M., Khanna A., Letchuman V., Letchuman S., Alexander J.S., Orr A.W., Woolard M.D., Pattillo C.B. (2018). Differential Arterial and Venous Endothelial Redox Responses to Oxidative Stress. Microcirculation.

[18] Song H.-H.G., Rumma R.T., Ozaki C.K., Edelman E.R., Chen C.S. (2018). Vascular Tissue Engineering: Progress, Challenges, and Clinical Promise. Cell Stem Cell.

[19] Camasão D.B., Mantovani D. (2021). The Mechanical Characterization of Blood Vessels and Their Substitutes in the Continuous Quest for Physiological-Relevant Performances. A Critical Review. Mater. Today Bio.

[20] Ercolani E., Del Gaudio C., Bianco A. (2015). Vascular Tissue Engineering of Small-Diameter Blood Vessels: Reviewing the Electrospinning Approach. J. Tissue Eng. Regen. Med..

[21] Mitchell R.N., Schoen F.J. (2010). Blood Vessels. Robbins and Cotran: Pathologic Basis of Disease.

[22] Zhang W.J., Liu W., Cui L., Cao Y. (2007). Tissue Engineering of Blood Vessel. J. Cell. Mol. Med..

[23] MacNeill B.D., Pomerantseva I., Lowe H.C., Oesterle S.N., Vacanti J.P. (2002). Toward a New Blood Vessel. Vasc. Med..

[24] Xu J., Shi G.-P. (2014). Vascular Wall Extracellular Matrix Proteins and Vascular Diseases. Biochim. Biophys. Acta.

[25] Cocciolone A.J., Hawes J.Z., Staiculescu M.C., Johnson E.O., Murshed M., Wagenseil J.E. (2018). Elastin, Arterial Mechanics, and Cardiovascular Disease. Am. J. Physiol. Heart Circ. Physiol..

[26] Awad N.K., Niu H., Ali U., Morsi Y.S., Lin T. (2018). Electrospun Fibrous Scaffolds for Small-Diameter Blood Vessels: A Review. Membranes.

[27] Enis I.Y., Sadikoglu T.G. (2018). Design Parameters for Electrospun Biodegradable Vascular Grafts. J. Ind. Text..

[28] Wang D., Xu Y., Li Q., Turng L.-S. (2020). Artificial Small-Diameter Blood Vessels: Materials, Fabrication, Surface Modification, Mechanical Properties, and Bioactive Functionalities. J. Mater. Chem. B.

[29] James B.D., Allen J.B. (2018). Vascular Endothelial Cell Behavior in Complex Mechanical Microenvironments. ACS Biomater. Sci. Eng..

[30] Zhang Y., Li X.S., Guex A.G., Liu S.S., Müller E., Malini R.I., Zhao H.J., Rottmar M., Maniura-Weber K., Rossi R.M. (2017). A Compliant and Biomimetic Three-Layered Vascular Graft for Small Blood Vessels. Biofabrication.

[31] Johnson R., Ding Y., Nagiah N., Monnet E., Tan W. (2019). Coaxially-Structured Fibres with Tailored Material Properties for Vascular Graft Implant. Mater. Sci. Eng. C Mater. Biol. Appl..

[32] Wu J., Hu C., Tang Z., Yu Q., Liu X., Chen H. (2018). Tissue-Engineered Vascular Grafts: Balance of the Four Major Requirements. Colloid Interface Sci. Commun..

[33] Hernandez J.L., Woodrow K.A. (2022). Medical Applications of Porous Biomaterials: Features of Porosity and Tissue-Specific Implications for Biocompatibility. Adv. Healthc. Mater..

[34] Kim G.H. (2008). Electrospun PCL Nanofibers with Anisotropic Mechanical Properties as a Biomedical Scaffold. Biomed. Mater..

[35] McFadden B.R., Smyth S.J. (2019). Perceptions of Genetically Engineered Technology in Developed Areas. Trends Biotechnol..

[36] Shariatzadeh S., Shafiee S., Zafari A., Tayebi T., Yazdanpanah G., Majd A., Haj-Mirzaian A., Bahrami S., Niknejad H. (2021). Developing a Pro-Angiogenic Placenta Derived Amniochorionic Scaffold with Two Exposed Basement Membranes as Substrates for Cultivating Endothelial Cells. Sci. Rep..

[37] Gao J., Chen S., Tang D., Jiang L., Shi J., Wang S. (2019). Mechanical Properties and Degradability of Electrospun PCL/PLGA Blended Scaffolds as Vascular Grafts. Trans. Tianjin Univ..

[38] Radke D., Jia W., Sharma D., Fena K., Wang G., Goldman J., Zhao F. (2018). Tissue Engineering at the Blood-Contacting Surface: A Review of Challenges and Strategies in Vascular Graft Development. Adv. Healthc. Mater..

[39] Obiweluozor F.O., Emechebe G.A., Kim D.-W., Cho H.-J., Park C.H., Kim C.S., Jeong I.S. (2020). Considerations in the Development of Small-Diameter Vascular Graft as an Alternative for Bypass and Reconstructive Surgeries: A Review. Cardiovasc. Eng. Technol..

[40] Eltom A., Zhong G., Muhammad A. (2019). Scaffold Techniques and Designs in Tissue Engineering Functions and Purposes: A Review. Adv. Mater. Sci. Eng..

[41] Zhao P., Gu H., Mi H., Rao C., Fu J., Turng L. (2018). Fabrication of Scaffolds in Tissue Engineering: A Review. Front. Mech. Eng..

[42] Li S., Sengupta D., Chien S. (2014). Vascular Tissue Engineering: From in Vitro to in Situ. Wiley Interdiscip. Rev. Syst. Biol. Med..

[43] Lu T., Li Y., Chen T. (2013). Techniques for Fabrication and Construction of Three-Dimensional Scaffolds for Tissue Engineering. Int. J. Nanomed..

[44] Kishan A.P., Cosgriff-Hernandez E.M. (2017). Recent Advancements in Electrospinning Design for Tissue Engineering Applications: A Review. J. Biomed. Mater. Res. A.

[45] Cui W., Zhou Y., Chang J. (2010). Electrospun Nanofibrous Materials for Tissue Engineering and Drug Delivery. Sci. Technol. Adv. Mater..

[46] Ahn H., Ju Y.M., Takahashi H., Williams D.F., Yoo J.J., Lee S.J., Okano T., Atala A. (2015). Engineered Small Diameter Vascular Grafts by Combining Cell Sheet Engineering and Electrospinning Technology. Acta Biomater..

[47] Raeisdasteh Hokmabad V., Davaran S., Ramazani A., Salehi R. (2017). Design and Fabrication of Porous Biodegradable Scaffolds: A Strategy for Tissue Engineering. J. Biomater. Sci. Polym. Ed..

[48] Yalcinkaya F. (2015). Experimental Study on Electrospun Polyvinyl Butyral Nanofibers Using a Non-Solvent System. Fibers Polym..

[49] Tan Z., Gao X., Liu T., Yang Y., Zhong J., Tong C., Tan Y. (2017). Electrospun Vein Grafts with High Cell Infiltration for Vascular Tissue Engineering. Mater. Sci. Eng. C Mater. Biol. Appl..

[50] Wang W., Nie W., Liu D., Du H., Zhou X., Chen L., Wang H., Mo X., Li L., He C. (2018). Macroporous Nanofibrous Vascular Scaffold with Improved Biodegradability and Smooth Muscle Cells Infiltration Prepared by Dual Phase Separation Technique. Int. J. Nanomed..

[51] Wang Z., Cui Y., Wang J., Yang X., Wu Y., Wang K., Gao X., Li D., Li Y., Zheng X.-L. (2014). The Effect of Thick Fibers and Large Pores of Electrospun Poly(ε-Caprolactone) Vascular Grafts on Macrophage Polarization and Arterial Regeneration. Biomaterials.

[52] O’Connor R.A., Cahill P.A., McGuinness G.B. (2021). Effect of Electrospinning Parameters on the Mechanical and Morphological Characteristics of Small Diameter PCL Tissue Engineered Blood Vessel Scaffolds Having Distinct Micro and Nano Fibre Populations—A DOE Approach. Polym. Test..

[53] Ju Y.M., Choi J.S., Atala A., Yoo J.J., Lee S.J. (2010). Bilayered Scaffold for Engineering Cellularized Blood Vessels. Biomaterials.

[54] Huang L., Guo S., Jiang Y., Shen Q., Li L., Shi Y., Xie H., Tian J. (2021). A Preliminary Study on Polycaprolactone and Gelatin-Based Bilayered Tubular Scaffolds with Hierarchical Pore Size Constructed from Nano and Microfibers for Vascular Tissue Engineering. J. Biomater. Sci. Polym. Ed..

[55] Woods I., Flanagan T.C. (2014). Electrospinning of Biomimetic Scaffolds for Tissue-Engineered Vascular Grafts: Threading the Path. Expert Rev. Cardiovasc. Ther..

[56] de Valence S., Tille J.-C., Giliberto J.-P., Mrowczynski W., Gurny R., Walpoth B.H., Möller M. (2012). Advantages of Bilayered Vascular Grafts for Surgical Applicability and Tissue Regeneration. Acta Biomater..

[57] Nottelet B., Pektok E., Mandracchia D., Tille J.-C., Walpoth B., Gurny R., Möller M. (2009). Factorial Design Optimization and in Vivo Feasibility of Poly (Epsilon-Caprolactone)-Micro- and Nanofiber-Based Small Diameter Vascular Grafts. J. Biomed. Mater. Res. A.

[58] Ratcliffe A. (2000). Tissue Engineering of Vascular Grafts. Matrix Biol..

[59] Guarino V., Causa F., Ambrosio L. (2007). Porosity and Mechanical Properties Relationship in PCL Porous Scaffolds. J. Appl. Biomater. Biomech..

[60] Ji S., Gu Q., Xia B. (2006). Porosity Dependence of Mechanical Properties of Solid Materials. J. Mater. Sci..

[61] Ang K.C., Leong K.F., Chua C.K., Chandrasekaran M. (2006). Investigation of the Mechanical Properties and Porosity Relationships in Fused Deposition Modelling-fabricated Porous Structures. Rapid Prototyp. J..

[62] Le Q.P., Uspenskaya M.V., Olekhnovich R.O., Baranov M.A. (2021). The Mechanical Properties of PVC Nanofiber Mats Obtained by Electrospinning. Fibers.

[63] Li Y., Lim C.T., Kotaki M. (2015). Study on Structural and Mechanical Properties of Porous PLA Nanofibers Electrospun by Channel-Based Electrospinning System. Polymer.

[64] Sarkar S., Salacinski H.J., Hamilton G., Seifalian A.M. (2006). The Mechanical Properties of Infrainguinal Vascular Bypass Grafts: Their Role in Influencing Patency. Eur. J. Vasc. Endovasc. Surg..

[65] Sarkar S., Hillery C., Seifalian A., Hamilton G. (2006). Critical Parameter of Burst Pressure Measurement in Development of Bypass Grafts Is Highly Dependent on Methodology Used. J. Vasc. Surg..

[66] Venugopal J., Vadgama P., Kumar T.S.S., Ramakrishna S. (2007). Biocomposite Nanofibres and Osteoblasts for Bone Tissue Engineering. Nanotechnology.

[67] Li L., Hashaikeh R., Arafat H.A. (2013). Development of Eco-Efficient Micro-Porous Membranes via Electrospinning and Annealing of Poly (Lactic Acid). J. Membr. Sci..

[68] de Valence S., Tille J.-C., Mugnai D., Mrowczynski W., Gurny R., Möller M., Walpoth B.H. (2012). Long Term Performance of Polycaprolactone Vascular Grafts in a Rat Abdominal Aorta Replacement Model. Biomaterials.

[69] Lovett M., Lee K., Edwards A., Kaplan D.L. (2009). Vascularization Strategies for Tissue Engineering. Tissue Eng Part B Rev.

[70] Putti M., Simonet M., Solberg R., Peters G.W.M. (2015). Electrospinning Poly (ε-Caprolactone) under Controlled Environmental Conditions: Influence on Fiber Morphology and Orientation. Polymer.

[71] Enis I.Y., Horakova J., Sadikoglu T.G., Novak O., Lukas D. (2017). Mechanical Investigation of Bilayer Vascular Grafts Electrospun from Aliphatic Polyesters. Polym. Adv. Technol..

[72] Rowland D.C.L., Aquilina T., Klein A., Hakimi O., Alexis-Mouthuy P., Carr A.J., Snelling S.J.B. (2016). A Comparative Evaluation of the Effect of Polymer Chemistry and Fiber Orientation on Mesenchymal Stem Cell Differentiation. J. Biomed. Mater. Res. A.

[73] Hasan A., Memic A., Annabi N., Hossain M., Paul A., Dokmeci M.R., Dehghani F., Khademhosseini A. (2014). Electrospun Scaffolds for Tissue Engineering of Vascular Grafts. Acta Biomater..

[74] Milleret V., Hefti T., Hall H., Vogel V., Eberli D. (2012). Influence of the Fiber Diameter and Surface Roughness of Electrospun Vascular Grafts on Blood Activation. Acta Biomater..

[75] Malik S., Sundarrajan S., Hussain T., Nazir A., Berto F., Ramakrishna S. (2021). Electrospun Biomimetic Polymer Nanofibers as Vascular Grafts. Mater. Des. Process. Commun..

[76] Murugan R., Ramakrishna S. (2007). Design Strategies of Tissue Engineering Scaffolds with Controlled Fiber Orientation. Tissue Eng..

[77] Wang Y., Wu T., Zhang J., Feng Z., Yin M., Mo X. (2021). A Bilayer Vascular Scaffold with Spatially Controlled Release of Growth Factors to Enhance in Situ Rapid Endothelialization and Smooth Muscle Regeneration. Mater. Des..

[78] Hu J.-J., Chao W.-C., Lee P.-Y., Huang C.-H. (2012). Construction and Characterization of an Electrospun Tubular Scaffold for Small-Diameter Tissue-Engineered Vascular Grafts: A Scaffold Membrane Approach. J. Mech. Behav. Biomed. Mater..

[79] Chaparro F.J., Matusicky M.E., Allen M.J., Lannutti J.J. (2016). Biomimetic Microstructural Reorganization during Suture Retention Strength Evaluation of Electrospun Vascular Scaffolds. J. Biomed. Mater. Res. Part B Appl. Biomater..

[80] Caves J.M., Kumar V.A., Martinez A.W., Kim J., Ripberger C.M., Haller C.A., Chaikof E.L. (2010). The Use of Microfiber Composites of Elastin-like Protein Matrix Reinforced with Synthetic Collagen in the Design of Vascular Grafts. Biomaterials.

[81] Nezarati R.M., Eifert M.B., Dempsey D.K., Cosgriff-Hernandez E. (2015). Electrospun Vascular Grafts with Improved Compliance Matching to Native Vessels. J. Biomed. Mater. Res. Part B Appl. Biomater..

[82] Yalcin I., Horakova J., Mikes P., Sadikoglu T.G., Domin R., Lukas D. (2016). Design of Polycaprolactone Vascular Grafts. J. Ind. Text..

[83] Gao J., Huang Z., Guo H., Tian S., Wang L., Li Y. (2019). Effect of Wall Structures on Mechanical Properties of Small Caliber PHBHHx Vascular Grafts. Fibers Polym..

[84] Meng X., Wang X., Jiang Y., Zhang B., Li K., Li Q. (2019). Suture Retention Strength of P (LLA-CL) Tissue-Engineered Vascular Grafts. RSC Adv..

[85] Jang B.S., Cheon J.Y., Kim S.H., Park W.H. (2018). Small Diameter Vascular Graft with Fibroblast Cells and Electrospun Poly (L-Lactide-Co-ε-Caprolactone) Scaffolds: Cell Matrix Engineering. J. Biomater. Sci. Polym. Ed..

[86] Wang C., Li Z., Zhang L., Sun W., Zhou J. (2020). Long-Term Results of Triple-Layered Small Diameter Vascular Grafts in Sheep Carotid Arteries. Med. Eng. Phys..

[87] Jeong Y., Yao Y., Yim E.K. (2020). Current Understanding of Intimal Hyperplasia and Effect of Compliance in Synthetic Small Diameter Vascular Grafts. Biomater. Sci..

[88] Bouchet M., Gauthier M., Maire M., Ajji A., Lerouge S. (2019). Towards Compliant Small-Diameter Vascular Grafts: Predictive Analytical Model and Experiments. Mater. Sci. Eng. C.

[89] Liu K., Wang N., Wang W., Shi L., Li H., Guo F., Zhang L., Kong L., Wang S., Zhao Y. (2017). A Bio-Inspired High Strength Three-Layer Nanofiber Vascular Graft with Structure Guided Cell Growth. J. Mater. Chem. B.

[90] Wu T., Zhang J., Wang Y., Li D., Sun B., El-Hamshary H., Yin M., Mo X. (2018). Fabrication and Preliminary Study of a Biomimetic Tri-Layer Tubular Graft Based on Fibers and Fiber Yarns for Vascular Tissue Engineering. Mater. Sci. Eng. C.

[91] Tolba E. (2020). Diversity of Electrospinning Approach for Vascular Implants: Multilayered Tubular Scaffolds. Regen. Eng. Transl. Med..

[92] Oztemur J., Yalcin Enis I. (2020). The Role of Biopolymer Selection in the Design of Electrospun Small Caliber Vascular Grafts to Replace the Native Arterial Structure. Chapter.

[93] Huang R., Gao X., Wang J., Chen H., Tong C., Tan Y., Tan Z. (2018). Triple-Layer Vascular Grafts Fabricated by Combined E-Jet 3D Printing and Electrospinning. Ann. Biomed. Eng..

[94] Grasl C., Stoiber M., Röhrich M., Moscato F., Bergmeister H., Schima H. (2021). Electrospinning of Small Diameter Vascular Grafts with Preferential Fiber Directions and Comparison of Their Mechanical Behavior with Native Rat Aortas. Mater. Sci. Eng. C.

[95] Ravi S., Chaikof E.L. (2010). Biomaterials for Vascular Tissue Engineering. Regen. Med..

[96] Wang Z., Liu L., Mithieux S.M., Weiss A.S. (2021). Fabricating Organized Elastin in Vascular Grafts. Trends Biotechnol..

[97] Zhu J., Chen D., Du J., Chen X., Wang J., Zhang H., Chen S., Wu J., Zhu T., Mo X. (2020). Mechanical Matching Nanofibrous Vascular Scaffold with Effective Anticoagulation for Vascular Tissue Engineering. Compos. Part B Eng..

[98] Yu E., Mi H.-Y., Zhang J., Thomson J.A., Turng L.-S. (2018). Development of Biomimetic Thermoplastic Polyurethane/Fibroin Small-Diameter Vascular Grafts via a Novel Electrospinning Approach. J. Biomed. Mater. Res. A.

[99] Ong C.S., Zhou X., Huang C.Y., Fukunishi T., Zhang H., Hibino N. (2017). Tissue Engineered Vascular Grafts: Current State of the Field. Expert Rev. Med. Devices.

[100] Szafron J.M., Khosravi R., Reinhardt J., Best C.A., Bersi M.R., Yi T., Breuer C.K., Humphrey J.D. (2018). Immuno-Driven and Mechano-Mediated Neotissue Formation in Tissue Engineered Vascular Grafts. Ann. Biomed. Eng..

[101] Pashneh-Tala S., MacNeil S., Claeyssens F. (2016). The Tissue-Engineered Vascular Graft-Past, Present, and Future. Tissue Eng. Part B Rev..

[102] Browning M.B., Dempsey D., Guiza V., Becerra S., Rivera J., Russell B., Höök M., Clubb F., Miller M., Fossum T. (2012). Multilayer Vascular Grafts Based on Collagen-Mimetic Proteins. Acta Biomater..

[103] Copes F., Pien N., Van Vlierberghe S., Boccafoschi F., Mantovani D. (2019). Collagen-Based Tissue Engineering Strategies for Vascular Medicine. Front. Bioeng. Biotechnol..

[104] Senthil R., Kavukcu S.B., Lakshmi T., Gülşah T., Candaş A.Z.A. (2022). Collagen/Physiologically Clotted Fibrin-Based Nanobioscaffold Supported with Silver Nanoparticles: A Novel Approach. Int. J. Artif. Organs.

[105] Antunes M., Bonani W., Reis R.L., Migliaresi C., Ferreira H., Motta A., Neves N.M. (2022). Development of Alginate-Based Hydrogels for Blood Vessel Engineering. Biomater. Adv..

[106] Gheorghita Puscaselu R., Lobiuc A., Dimian M., Covasa M. (2020). Alginate: From Food Industry to Biomedical Applications and Management of Metabolic Disorders. Polymers.

[107] Sahoo D.R., Biswal T. (2021). Alginate and Its Application to Tissue Engineering. SN Appl. Sci..

[108] Ahsan S.M., Thomas M., Reddy K.K., Sooraparaju S.G., Asthana A., Bhatnagar I. (2018). Chitosan as Biomaterial in Drug Delivery and Tissue Engineering. Int. J. Biol. Macromol..

[109] Croisier F., Jérôme C. (2013). Chitosan-Based Biomaterials for Tissue Engineering. Eur. Polym. J..

[110] Islam M.M., Shahruzzaman M., Biswas S., Nurus Sakib M., Rashid T.U. (2020). Chitosan Based Bioactive Materials in Tissue Engineering Applications-A Review. Bioact. Mater..

[111] Foster J.A., Lennarz W.J., Lane M.D. (2013). Elastin. Encyclopedia of Biological Chemistry.

[112] Gomes M., Azevedo H., Malafaya P., Silva S., Oliveira J., Silva G., Sousa R., Mano J., Reis R., van Blitterswijk C., Thomsen P., Lindahl A., Hubbell J., Williams D.F., Cancedda R., de Bruijn J.D., Sohier J. (2008). Chapter 6—Natural Polymers in Tissue Engineering Applications. Tissue Engineering.

[113] Nasrollahzadeh M., Maham M., Nezafat Z., Shafiei N., Nasrollahzadeh M. (2021). Chapter 4—Protein and Polypeptide Biopolymer Chemistry. Biopolymer-Based Metal Nanoparticle Chemistry for Sustainable Applications.

[114] Janmey P.A., Winer J.P., Weisel J.W. (2009). Fibrin Gels and Their Clinical and Bioengineering Applications. J. R. Soc. Interface.

[115] Liu R.H., Ong C.S., Fukunishi T., Ong K., Hibino N. (2018). Review of Vascular Graft Studies in Large Animal Models. Tissue Eng. Part B Rev..

[116] Shaikh F.M., Callanan A., Kavanagh E.G., Burke P.E., Grace P.A., McGloughlin T.M. (2008). Fibrin: A Natural Biodegradable Scaffold in Vascular Tissue Engineering. Cells Tissues Organs.

[117] Sundararaghavan H.G., Burdick J.A., Ducheyne P. (2011). 5.509—Cell Encapsulation. Comprehensive Biomaterials.

[118] Aldana A.A., Abraham G.A. (2017). Current Advances in Electrospun Gelatin-Based Scaffolds for Tissue Engineering Applications. Int. J. Pharm..

[119] Asadpour S., Kargozar S., Moradi L., Ai A., Nosrati H., Ai J. (2020). Natural Biomacromolecule Based Composite Scaffolds from Silk Fibroin, Gelatin and Chitosan toward Tissue Engineering Applications. Int. J. Biol. Macromol..

[120] Deshmukh K., Basheer Ahamed M., Deshmukh R.R., Khadheer Pasha S.K., Bhagat P.R., Chidambaram K., Sadasivuni K.K., Ponnamma D., Kim J., Cabibihan J.-J., AlMaadeed M.A. (2017). 3—Biopolymer Composites with High Dielectric Performance: Interface Engineering. Biopolymer Composites in Electronics.

[121] McKeen L., McKeen L. (2021). Chapter11—The Effect of Heat Aging on the Properties of Sustainable Polymers. The Effect of Long Term Thermal Exposure on Plastics and Elastomers.

[122] Mohamed R.M., Yusoh K. (2016). A Review on the Recent Research of Polycaprolactone (PCL). Adv. Mater. Res..

[123] Patrício T., Domingos M., Gloria A., Bártolo P. (2013). Characterisation of PCL and PCL/PLA Scaffolds for Tissue Engineering. Procedia CIRP.

[124] Pavia F.C., Rigogliuso S., Carrubba V.L., Mannella G.L., Ghersi G., Brucato V. (2012). Poly Lactic Acid Based Scaffolds for Vascular Tissue Engineering. Chem. Eng. Trans..

[125] Donate R., Monzón M., Alemán-Domínguez M.E. (2020). Additive Manufacturing of PLA-Based Scaffolds Intended for Bone Regeneration and Strategies to Improve Their Biological Properties. e-Polymers.

[126] Santoro M., Shah S.R., Walker J.L., Mikos A.G. (2016). Poly(Lactic Acid) Nanofibrous Scaffolds for Tissue Engineering. Adv. Drug Deliv. Rev..

[127] Yazdanpanah A., Amoabediny G., Shariatpanahi P., Nourmohammadi J., Tahmasbi M., Mozafari M. (2014). Synthesis and Characterization of Polylactic Acid Tubular Scaffolds with Improved Mechanical Properties for Vascular Tissue Engineering. Trends Biomater. Artif. Organs.

[128] Budak K., Sogut O., Sezer U.A. (2020). A Review on Synthesis and Biomedical Applications of Polyglycolic Acid. J. Polym. Res..

[129] Hajiali H., Shahgasempour S., Naimi-Jamal M.R., Peirovi H. (2011). Electrospun PGA/Gelatin Nanofibrous Scaffolds and Their Potential Application in Vascular Tissue Engineering. Int. J. Nanomed..

[130] Thomas L.V., Lekshmi V., Nair P.D. (2013). Tissue Engineered Vascular Grafts—Preclinical Aspects. Int. J. Cardiol..

[131] Montini-Ballarin F., Calvo D., Caracciolo P.C., Rojo F., Frontini P.M., Abraham G.A., Guinea G.V. (2016). Mechanical Behavior of Bilayered Small-Diameter Nanofibrous Structures as Biomimetic Vascular Grafts. J. Mech. Behav. Biomed. Mater..

[132] J-Shaped Curves. https://www.doitpoms.ac.uk/tlplib/bioelasticity/j-shaped-curves.php.

[133] Benrashid E., McCoy C.C., Youngwirth L.M., Kim J., Manson R.J., Otto J.C., Lawson J.H. (2016). Tissue Engineered Vascular Grafts: Origins, Development, and Current Strategies for Clinical Application. Methods.

[134] Ryan A.J., Ryan E.J., Cameron A.R., O’Brien F.J. (2020). Hierarchical Biofabrication of Biomimetic Collagen-Elastin Vascular Grafts with Controllable Properties via Lyophilisation. Acta Biomater..

[135] Reddy M.S.B., Ponnamma D., Choudhary R., Sadasivuni K.K. (2021). A Comparative Review of Natural and Synthetic Biopolymer Composite Scaffolds. Polymers.

[136] Gupta P., Mandal B.B. (2021). Tissue-Engineered Vascular Grafts: Emerging Trends and Technologies. Adv. Funct. Mater..

[137] Park S., Kim J., Lee M.-K., Park C., Jung H.-D., Kim H.-E., Jang T.-S. (2019). Fabrication of Strong, Bioactive Vascular Grafts with PCL/Collagen and PCL/Silica Bilayers for Small-Diameter Vascular Applications. Mater. Des..

[138] Seifu D.G., Purnama A., Mequanint K., Mantovani D. (2013). Small-Diameter Vascular Tissue Engineering. Nat. Rev. Cardiol..

[139] Qiu Y., Myers D.R., Lam W.A. (2019). The Biophysics and Mechanics of Blood from a Materials Perspective. Nat. Rev. Mater..

[140] van Haaften E.E., Bouten C.V.C., Kurniawan N.A. (2017). Vascular Mechanobiology: Towards Control of In Situ Regeneration. Cells.

[141] Isenberg B.C., Williams C., Tranquillo R.T. (2006). Small-Diameter Artificial Arteries Engineered in Vitro. Circ. Res..

[142] Serbo J.V., Gerecht S. (2013). Vascular Tissue Engineering: Biodegradable Scaffold Platforms to Promote Angiogenesis. Stem Cell Res. Ther..

[143] Chiu J.-J., Chien S. (2011). Effects of Disturbed Flow on Vascular Endothelium: Pathophysiological Basis and Clinical Perspectives. Physiol. Rev..

[144] Zhou T., Zheng Y., Qiu J., Hu J., Sun D., Tang C., Wang G. (2014). Endothelial Mechanotransduction Mechanisms for Vascular Physiology and Atherosclerosis. J. Mech. Med. Biol..

[145] de Mel A., Murad F., Seifalian A.M. (2011). Nitric Oxide: A Guardian for Vascular Grafts?. Chem. Rev..

[146] Li Y.-S.J., Haga J.H., Chien S. (2005). Molecular Basis of the Effects of Shear Stress on Vascular Endothelial Cells. J. Biomech..

[147] Yamamoto K., Takahashi T., Asahara T., Ohura N., Sokabe T., Kamiya A., Ando J. (2003). Proliferation, Differentiation, and Tube Formation by Endothelial Progenitor Cells in Response to Shear Stress. J. Appl. Physiol..

[148] Mitchell S.L., Niklason L.E. (2003). Requirements for Growing Tissue-Engineered Vascular Grafts. Cardiovasc. Pathol..

[149] Dan P., Velot É., Decot V., Menu P. (2015). The Role of Mechanical Stimuli in the Vascular Differentiation of Mesenchymal Stem Cells. J. Cell Sci..

[150] Jufri N.F., Mohamedali A., Avolio A., Baker M.S. (2015). Mechanical Stretch: Physiological and Pathological Implications for Human Vascular Endothelial Cells. Vasc. Cell.

[151] Kwak B.R., Bäck M., Bochaton-Piallat M.-L., Caligiuri G., Daemen M.J.A.P., Davies P.F., Hoefer I.E., Holvoet P., Jo H., Krams R. (2014). Biomechanical Factors in Atherosclerosis: Mechanisms and Clinical Implications. Eur. Heart J..

[152] Green D.J., Hopman M.T.E., Padilla J., Laughlin M.H., Thijssen D.H.J. (2017). Vascular Adaptation to Exercise in Humans: Role of Hemodynamic Stimuli. Physiol. Rev..

[153] Castillo-Cruz O., Pérez-Aranda C., Gamboa F., Cauich-Rodríguez J.V., Mantovani D., Avilés F. (2018). Prediction of Circumferential Compliance and Burst Strength of Polymeric Vascular Grafts. J. Mech. Behav. Biomed. Mater..

[154] Castorena-Gonzalez J.A., Staiculescu M.C., Foote C., Martinez-Lemus L.A. (2014). Mechanisms of the Inward Remodeling Process in Resistance Vessels: Is the Actin Cytoskeleton Involved?. Microcirculation.

[155] Bersi M.R., Bellini C., Humphrey J.D., Avril S. (2019). Local Variations in Material and Structural Properties Characterize Murine Thoracic Aortic Aneurysm Mechanics. Biomech. Model. Mechanobiol..

[156] Sanft R., Power A., Nicholson C. (2019). Modeling the Effects of Muscle Contraction on the Mechanical Response and Circumferential Stability of Coronary Arteries. Math. Biosci..

[157] Thubrikar M.J. (2007). Pressure Vessel Principles. Vascular Mechanics and Pathology.

[158] Ferreira H.P., Moura D., Pereira A.T., Henriques P.C., Barrias C.C., Magalhães F.D., Gonçalves I.C. (2022). Using Graphene-Based Materials for Stiff and Strong Poly(Ethylene Glycol) Hydrogels. Int. J. Mol. Sci..

[159] Olsen T.R., Casco M., Herbst A., Evans G., Rothermel T., Pruett L., Reid J., Barry K., Jaeggli M.P., Simionescu D.T. (2016). Longitudinal Stretching for Maturation of Vascular Tissues Using Magnetic Forces. Bioengineering.

[160] Jackson Z.S., Gotlieb A.I., Langille B.L. (2002). Wall Tissue Remodeling Regulates Longitudinal Tension in Arteries. Circ. Res..

[161] Han H.-C., Ku D.N., Vito R.P. (2003). Arterial Wall Adaptation under Elevated Longitudinal Stretch in Organ Culture. Ann. Biomed. Eng..

[162] Elliott M.B., Gerecht S. (2016). Three-Dimensional Culture of Small-Diameter Vascular Grafts. J. Mater. Chem. B.

[163] Das A., Paul A., Taylor M.D., Banerjee R.K. (2015). Pulsatile Arterial Wall-Blood Flow Interaction with Wall Pre-Stress Computed Using an Inverse Algorithm. BioMed. Eng. OnLine.

[164] Chaouat M., Le Visage C., Baille W.E., Escoubet B., Chaubet F., Mateescu M.A., Letourneur D. (2008). A Novel Cross-linked Poly (Vinyl Alcohol)(PVA) for Vascular Grafts. Adv. Funct. Mater..

[165] Johnson J., Ohst D., Groehl T., Hetterscheidt S., Jones M. (2015). Development of Novel, Bioresorbable, Small-Diameter Electrospun Vascular Grafts. J. Tissue Sci. Eng..

[166] Greenwald S., Berry C. (2000). Improving Vascular Grafts: The Importance of Mechanical and Haemodynamic Properties. J. Pathol..

[167] Inoue T., Kanda K., Yamanami M., Kami D., Gojo S., Yaku H. (2021). Modifications of the Mechanical Properties of in Vivo Tissue-Engineered Vascular Grafts by Chemical Treatments for a Short Duration. PLoS ONE.

[168] Marinov G., Guidoin R., Tse L.W., Ruthrauff A.A., Yao T., King M.W., King M.W., Gupta B.S., Guidoin R. (2013). 21—Endovascular Prostheses for Aortic Aneurysms: A New Era for Vascular Surgery. Biotextiles as Medical Implants.

[169] Rapoport H.S., Fish J., Basu J., Campbell J., Genheimer C., Payne R., Jain D. (2012). Construction of a Tubular Scaffold That Mimics J-Shaped Stress/Strain Mechanics Using an Innovative Electrospinning Technique. Tissue Eng. Part C Methods.

[170] Akentjew T.L., Terraza C., Suazo C., Maksimcuka J., Wilkens C.A., Vargas F., Zavala G., Ocaña M., Enrione J., García-Herrera C.M. (2019). Rapid Fabrication of Reinforced and Cell-Laden Vascular Grafts Structurally Inspired by Human Coronary Arteries. Nat. Commun..

[171] Kim S.-H., Mun C.H., Jung Y., Kim S.-H., Kim D.-I., Kim S.H. (2013). Mechanical Properties of Compliant Double Layered Poly (L-Lactide-Co-ɛ-Caprolactone) Vascular Graft. Macromol. Res..

[172] Drilling S., Gaumer J., Lannutti J. (2009). Fabrication of Burst Pressure Competent Vascular Grafts via Electrospinning: Effects of Microstructure. J. Biomed. Mater. Res. Part A.

[173] Montini-Ballarin F., Abraham G., Caracciolo P. (2016). Mechanical Behavior of Polyurethane-Based Small-Diameter Vascular Grafts. Advances in Polyurethane Biomaterials.

[174] Post A., Diaz-Rodriguez P., Balouch B., Paulsen S., Wu S., Miller J., Hahn M., Cosgriff-Hernandez E. (2019). Elucidating the Role of Graft Compliance Mismatch on Intimal Hyperplasia Using an Ex Vivo Organ Culture Model. Acta Biomater..

[175] Goonoo N., Bhaw-Luximon A., Bowlin G.L., Jhurry D. (2013). An Assessment of Biopolymer-and Synthetic Polymer-based Scaffolds for Bone and Vascular Tissue Engineering. Polym. Int..

[176] He F., Hua L., Gao L. (2015). A Computational Model for Biomechanical Effects of Arterial Compliance Mismatch. Appl. Bionics Biomech..

[177] Wise S.G., Byrom M.J., Waterhouse A., Bannon P.G., Ng M.K., Weiss A.S. (2011). A Multilayered Synthetic Human Elastin/Polycaprolactone Hybrid Vascular Graft with Tailored Mechanical Properties. Acta Biomater..

[178] Spadaccio C., Nappi F., Al-Attar N., Sutherland F.W., Acar C., Nenna A., Trombetta M., Chello M., Rainer A. (2016). Old Myths, New Concerns: The Long-Term Effects of Ascending Aorta Replacement with Dacron Grafts. Not All That Glitters Is Gold. J. Cardiovasc. Trans. Res..

[179] Matsuzaki Y., Iwaki R., Reinhardt J.W., Chang Y.-C., Miyamoto S., Kelly J., Zbinden J., Blum K., Mirhaidari G., Ulziibayar A. (2020). The Effect of Pore Diameter on Neo-Tissue Formation in Electrospun Biodegradable Tissue-Engineered Arterial Grafts in a Large Animal Model. Acta Biomater..

[180] Bazgir M., Zhang W., Zhang X., Elies J., Saeinasab M., Coates P., Youseffi M., Sefat F. (2021). Degradation and Characterisation of Electrospun Polycaprolactone (PCL) and Poly (Lactic-Co-Glycolic Acid)(PLGA) Scaffolds for Vascular Tissue Engineering. Materials.

[181] Yang L., Li X., Wang D., Mu S., Lv W., Hao Y., Lu X., Zhang G., Nan W., Chen H. (2020). Improved Mechanical Properties by Modifying Fibrin Scaffold with PCL and Its Biocompatibility Evaluation. J. Biomater. Sci. Polym. Ed..

[182] Bolbasov E., Goreninskii S., Tverdokhlebov S., Mishanin A., Viknianshchuk A., Bezuidenhout D., Golovkin A. (2018). Comparative Study of the Physical, Topographical and Biological Properties of Electrospinning PCL, PLLA, Their Blend and Copolymer Scaffolds. IOP Conf. Ser. Mater. Sci. Eng..

